# Discovering Heterogeneous Leukocytes Subsets Associated With Alcoholic Steatohepatitis by scRNAseq Analysis

**DOI:** 10.1002/mco2.70448

**Published:** 2025-11-02

**Authors:** Haribalan Perumalsamy, Sehee Park, Ji Eun Kim, Xiao Xiao, Hye Young Kim, Dae Won Jun, Tae‐Hyun Yoon

**Affiliations:** ^1^ Research Institute for Convergence of Basic Science Hanyang University Seongdong‐gu Seoul Republic of Korea; ^2^ Center For Creative Convergence Education Hanyang University Seongdong‐gu Seoul Republic of Korea; ^3^ Department of Chemistry College of Natural Sciences Hanyang University Seongdong‐gu Seoul Republic of Korea; ^4^ Department of Translational Medicine Graduate School of Biomedical Science and Engineering Hanyang University Seongdong‐gu Seoul Republic of Korea; ^5^ Hanyang Institute of Bioscience and Biotechnology Hanyang University Seoul Republic of Korea; ^6^ Department of Internal Medicine College of Medicine Hanyang University Seongdong‐gu Seoul Republic of Korea; ^7^ Institute For Next‐Generation Material Design Hanyang University Seongdong‐gu Seoul Republic of Korea; ^8^ Yoon Idea Lab. Co. Ltd Seoul Republic of Korea

**Keywords:** alcoholic liver disease, alcoholic steatohepatitis, B cell, circulating immune cells, neutrophils, scRNAseq, tSNEs

## Abstract

The precise identification of immune cell type responses to alcoholic steatohepatitis (ASH) at the single‐cell level remains unresolved. Therefore, in this study, we analyzed heterogeneous immune leukocytes associated with ASH at the single‐cell level using high‐dimensional single‐cell RNA sequencing in alcoholic liver disease (ALD)‐induced and healthy control mice. A t‐distributed stochastic neighbor embedding plot for dimensionality reduction and 2D visualization was used to visualize heterogeneous immune cell types. Moreover, singleR was used for automated cell annotation to identify the cell types and differentially expressed genes from each cell type and their subsets. We observed a decline in the population of B cells and their subsets, with up and downregulated genes signifying an innate proinflammatory response as an important indication of alcohol‐induced liver fibrosis. Additionally, neutrophil deficiency in the alcohol‐induced mouse group was associated with ASH. An increase in eosinophils diverts further complications in liver fibrosis, suggesting the functional heterogeneity of granulocyte subsets. Overall, our findings may assist in discovering potential ALD biomarker cell types that are significantly reduced by frequent alcohol exposure and enhance our understanding of the circulating immune leukocytes that lead to alcohol‐induced liver fibrosis.

## Graphical abstract

In this study, we explored comprehensive profiling of heterogeneous immune leukocytes at a single‐cell level using scRNAseq analysis to discover the regulation of inflammatory‐mediated liver fibrosis in ALD‐induced mice compared with healthy controls.

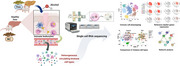

## 1 Introduction

Chronic alcohol consumption contributes to alcoholic steatohepatitis (ASH), which is characterized by hepatocellular liver injury caused by infiltrated immune cells and can lead to fibrosis, cirrhosis, and hepatocellular carcinoma [1, 2, 3]. However, chronic alcohol consumption causes carcinogenic effects not only in the liver as hepatotoxic, but also in other extrahepatic organs such as the gut, spleen, lungs, breast, and colorectal system. Since most blood flow to the liver (over 80%) travels through extrahepatic organs, exposing it to pathogenic antigens on a regular basis, gut‐derived bacterial‐mediated toxins and their environmental contamination may cause a hyper‐ or hypo‐immune response to continue defending the liver from those pathogenic agents [4, 5]. Interorgan crosstalk involving the gut, liver, adipose tissue, muscle, lungs, and the neuroendocrine system likely plays a role in the inflammation of alcoholic liver disease (ALD) and has been studied extensively [6, 7, 8]. However, the immune cell types and inflammatory responses in the liver associated with crosstalk of other organs in response to ALD is poorly understood at the single‐cell level [9, 10, 11].

The circulation of immune cells through the bloodstream may be responsible for pathogenic microbe infiltration in the liver, which leads to an inflammatory response in ALD patients, causing severe liver damage [12]. Though many review articles have been written about the importance of liver lymphocytes, microbial infiltration, and immunological responses in fibrotic changes in the liver [10, 11], it is challenging to identify early signs of liver inflammation through biopsy [6, 12, 13, 14, 15], unless immunological reactions are triggered by pathogenic chemicals from multiple sources.

Recent evidence suggests that immune cells play a crucial role in ALD through induction of the inflammatory response. During inflammation, immune cells such as neutrophils, Kupffer cells, natural killer cells (NK), and NK T cells respond to danger signals including pathogen‐associated molecular patterns (produced mainly by the gut microbiome) and damage‐associated molecular patterns (released by damaged cells) through surface receptors by initiating cellular activation and intracellular signal transduction processes [16, 17, 18, 19]. This results in activation of various inflammatory cascades, which contribute to the severity of the ALD [12, 15].

Despite extensive research on ASH, early signs of liver inflammation caused by alcoholic byproducts or pathogenic chemicals are difficult to detect and are frequently inaccurate. For example, homogeneous bulk‐RNA sequencing may lead to misconceptions due to its inability to identify immune cell types in heterogeneous environments and may misinterpret transcriptomic expression in the complex cellular environments underlying the pathogenesis of ALD [20]. Therefore, assessing immune cell type heterogeneity and transcriptomic profiling of expressed genes from lymphocytes of interest at a single level could provide a comprehensive understanding of the complexity of immune cell types [21, 22, 23]. The functional diversity and transcriptomic responses of heterogeneous immune cell types from ALD are not well known. Recently, Cao et al. [20, 21, 22] used single‐cell RNA sequencing (scRNAseq) to identify resident heterogeneous liver cell subsets that are primarily affected by short‐term alcoholic liver. However, limited research focuses on circulating immune cells., which may be responsible for pathogenic microbe infiltration in the liver, resulting in severe liver fibrosis [24]. It is also believed that circulating immune cells may influence interorgan crosstalk and play a role in the development of ALD inflammation [25].

Therefore, in our study, we explored comprehensive profiling of heterogeneous immune leukocytes at a single‐cell level using scRNAseq analysis to discover the regulation of inflammatory‐mediated liver fibrosis in ALD‐induced mice compared with healthy controls. We demonstrate that a substantial innate inflammatory response from circulating immune cell types, such as B cells, granulocytes, monocytes, and their subsets, influence alcohol‐induced liver fibrosis through infiltration. Furthermore, transcriptomic profiling of cell types and liver tissue revealed differentially expressed genes (DEGs), functional enrichment, and their molecular pathways. Our findings could contribute to a better understanding of the circulating immune leukocytes that cause alcohol‐induced liver fibrosis and assist in the identification of potential ALD biomarkers.

## 2 Results

### 2.1 Preprocessing of scRNAseq and Bulk‐RNA Data Processing

Cell Ranger was used to process the fastq files of datasets for the mouse groups. During the preprocessing steps, we excluded low‐quality genes by setting gene numbers <500 or >6000 throughout the preprocessing phases. The detailed information is provided in Figure S1.

### 2.2 ALD‐Induced Mice Presented Liver Injury and Increased Liver Markers

**FIGURE 1:**
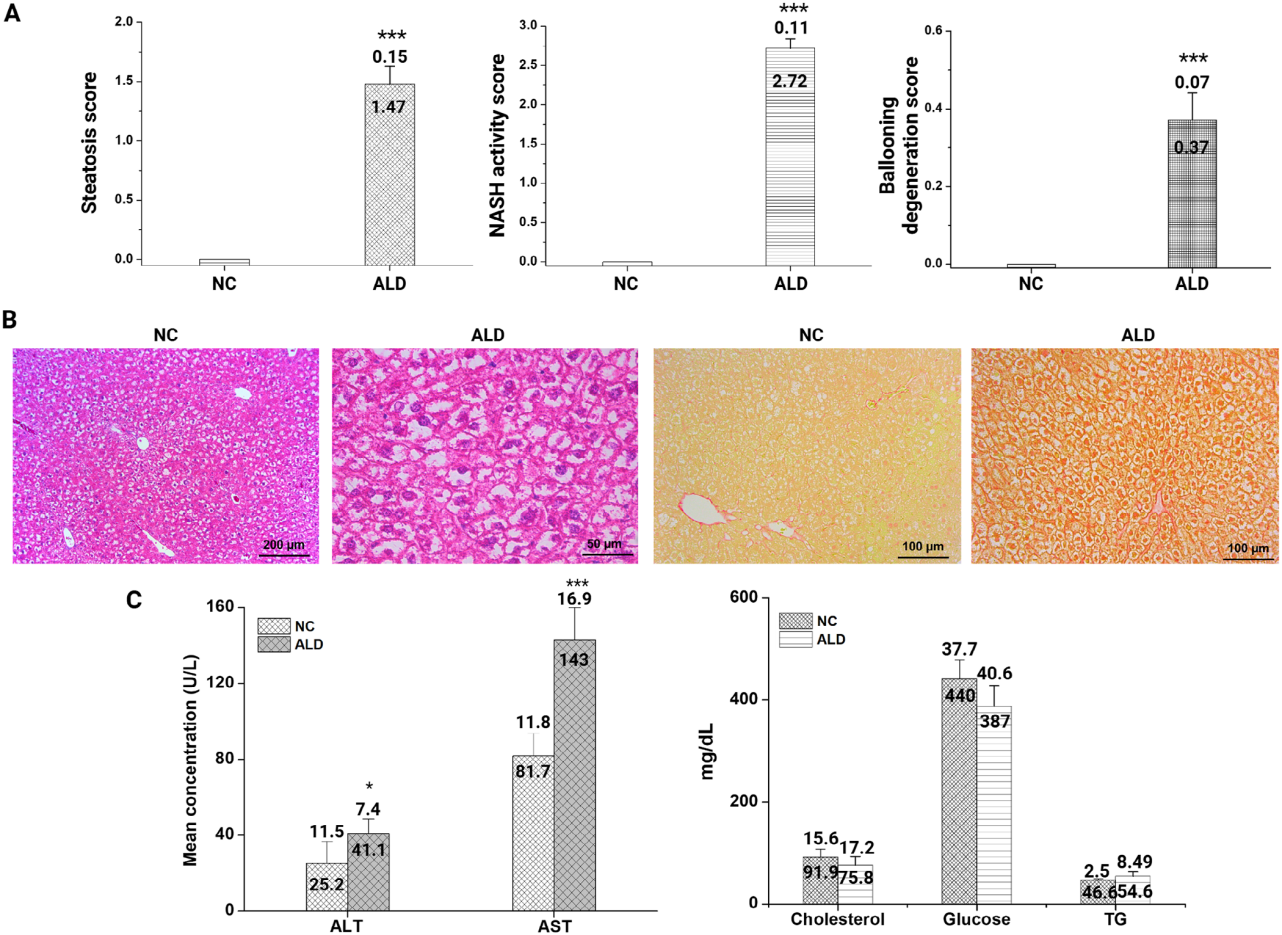
Assessment of liver pathology in a mouse model. (A) Liver pathology scores across NC and ALD groups. Scores are presented for steatosis, nonalcoholic steatohepatitis (NASH) activity, and ballooning degeneration. (B) Histological analysis of liver sections from NC and ALD mouse models. Hematoxylin and eosin (H&E) and Sirius staining reveal cellular and structural changes in the liver tissue across groups. (C) Biochemical parameters of blood samples collected from ALD and NC mice were validated and compared.

After exposure to an alcoholic diet for 17 days, the fibrosis effect in liver tissue was confirmed by histochemistry with hematoxylin and eosin (H&E) and Sirius. The staining prominently identified fibrotic changes with higher levels of disintegrated enlarged cells in ALD‐induced mice compared with NC mice (Figure 1A,B). Histochemical scoring of steatosis, NASH activity, and ballooning degeneration revealed that ALD‐induced mice groups were more prone to liver damage than the NC group (Figure 1A). The H&E and Sirius staining clearly identified liver injury following persistent exposure to an alcoholic diet (Figure 1B).

Biochemical analysis was conducted on blood samples collected from the mouse groups. Alanine transaminase (ALT) and aspartate transferase (AST) are key liver enzymes that act as markers of liver health as they are released when liver cells are damaged. The high levels of both ALT and AST in the ALD‐induced mouse group denote significant liver damage (Figure 1C). Additionally, glucose and lipid metabolism in a healthy liver play an important role in energy storage, and malfunction was observed in ALD‐induced mice (Figure 1C).

### 2.3 Immune Leukocytes are Associated with ALD

**FIGURE 2:**
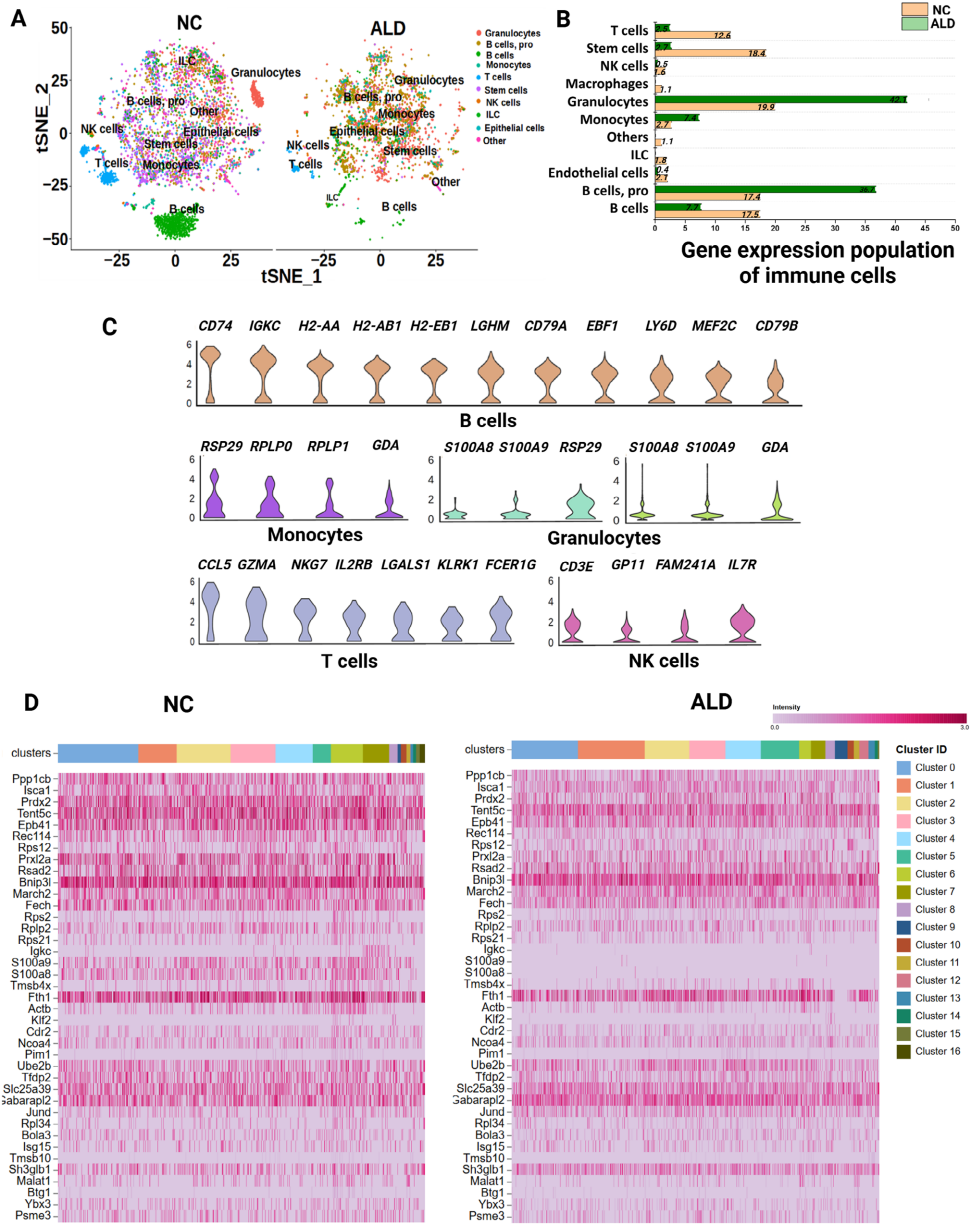
Identification of heterogeneous immune cell populations by scRNAseq analysis. (A) t‐SNE visualization of immune cell types in both NC and ALD‐induced mouse groups. (B) Population differences of identified immune leukocytes from the NC and ALD‐induced mouse groups. (C) List of reference marker genes from each significant immune cell type. (D) Heat map expression comparison of overall markers genes from all cell types in NC and ALD‐induced mouse groups. Statistically significant differences at 0.01 < *p* < 0.05, 0.001 < *p* < 0.01, and *p *< 0.001 are annotated as *, **, and ***, respectively.

This section provides an in‐depth analysis of population differences and gene expression profiles in both the NC and ALD‐induced mouse groups (Figure 2). Figure 2A provides a visualization of the comprehensive scRNAseq data analysis in a high‐dimensional tSNE plot. Heterogeneous immune cell types were identified, and distinct cell types were specified, each with its own set of expression marker genes. The predominant cell types were identified as neutrophils, premature B cells (pro‐B cells), B cells, monocytes, T cells, stem cells, NK cells, eosinophils, innate lymphoid cells, epithelial cells, and other minor cell types (Figure 2A). In general, the ALD‐induced mouse group showed distinct population changes from each cell type compared with the NC group. Specifically, significant population reduction was observed in B, T, stem, and NK cells. However, neutrophils, pro‐B cells, and monocytes were increased in the ALD‐induced mice (Figure 2B). These findings indicated immunological dysfunction in ALD via inflammatory mediated‐heterogeneous responses of immune cell types. To determine the cell types responsible for the inflammatory response from both ALD and NC groups, the expression patterns of unique reference marker genes (Figure S2) were examined. Figure 2C shows the significant reference gene markers from each cell type. For B cells, 11 marker genes were expressed, *Cd74*, *Igkc*, *H2‐Aa*, *H2‐Ab1*, *H2‐Eb1*, *Ihgm*, *Cd79a*, *Ebf1*, *Ly6d*, *Mef2c*, and *Cd79b*. Monocytes expressed four marker genes (*Rps29*, *Rplp0*, *Rplp1*, and *Gda*), granulocytes expressed four genes (*S100a8*, *S100a9*, *Gda*, and *Rps29*), NK cells expressed seven genes (*Ccl5*, *Gzma*, *Nkg7*, *Il2rb*, *Lgals1*, *Klrk1*, and *Fcer1g*), and T cells expressed four genes (*Cd3e*, *Gpi1*, *Fam241a*, and *Il7γ*). The cellular gene profiles of total heterogeneous immune leukocytes from NC and ALD‐induced mouse groups are shown in Figure 2D. Significant gene expression was observed in B cells, granulocytes, monocytes, NK cells, and T cells. Our study revealed significant patterns of expression in specific immune cell types in the ALD‐induced mice compared with the control mice. Many studies have focused on identifying the immune cell population that is responsible for ALD. However, complete in‐depth profiling of liver leukocytes has received less attention, and there is a necessity to understand the molecular pathways and genes associated with ALD.

### 2.4 Transitional, Follicular, and Mature B Cells are Involved in ALD

**FIGURE 3:**
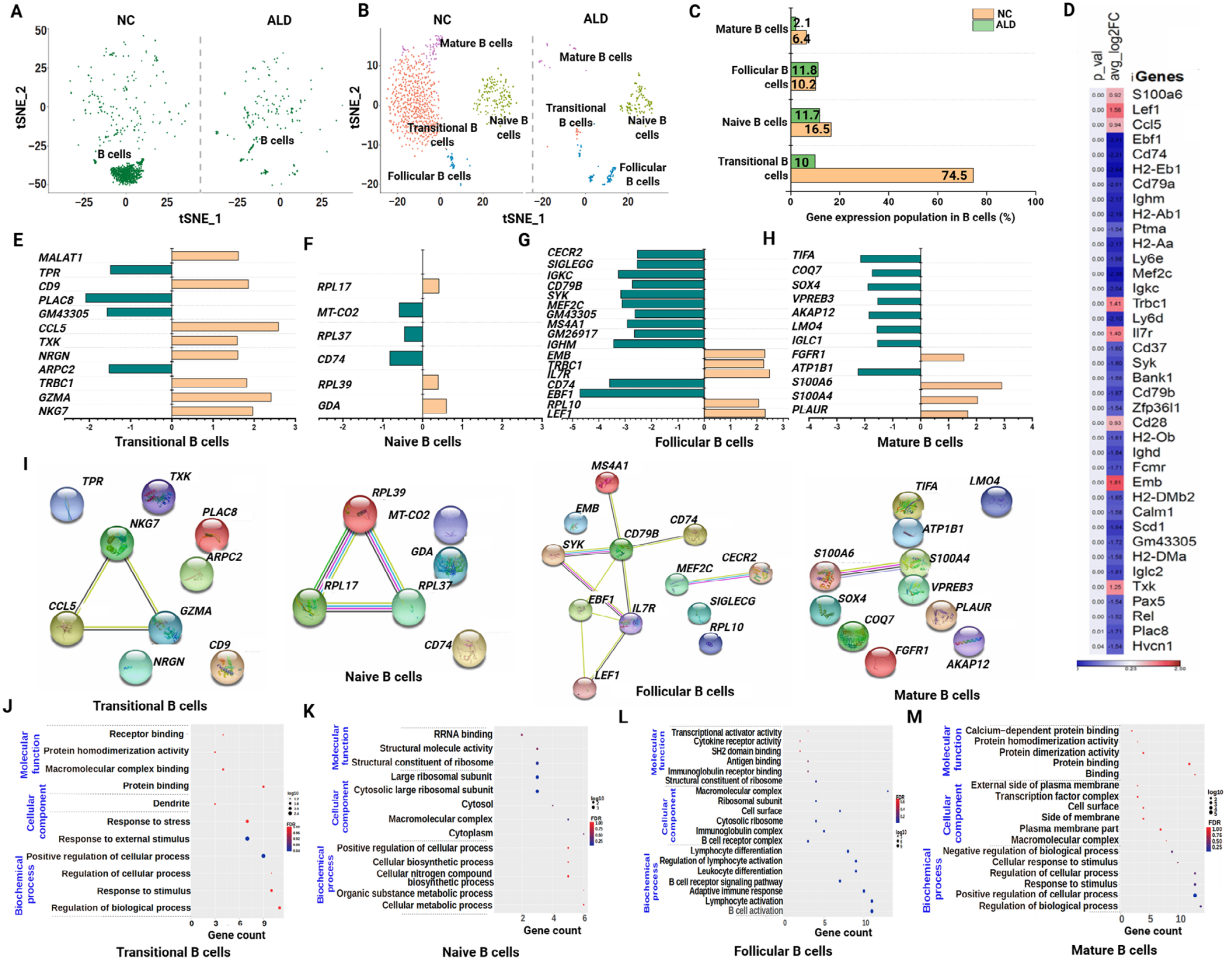
Deep immunological profiling of B cells. (A) tSNE visualization of total B cells from NC and ALD‐induced mouse groups. (B) Identification of B cell subsets by the PhenoGraph clustering algorithm. (C) Population differences of B cell subsets from NC and ALD‐induced mice. (D) Overall heat map expression of DEGs from B cells. (E–H) The DEGs of each B cell subset were depicted as a bar graph representing up‐ or downregulation. (I) Network profiling analysis of the B cell subset with DEG in NC and ALD‐induced mice groups. (J–M) GO profiling of differentially expressed genes from B cell subsets showing gene count with false discovery rate (FDR) and log10, *p* value. Statistically significant differences at 0.01 < *p* < 0.05, 0.001 <* p* < 0.01, and *p* < 0.001 have been annotated as *, **, and ***, respectively.

The scRNAseq analysis revealed a significant reduction in the population of B cells in the ALD‐induced mice (Figure 2B). However, the reason behind the changes in the B cell population remains undetermined. Comprehensive profiling was performed to visualize the B cell subset population and their gene expression profiles. A PhenoGraph (PG) clustering algorithm method was used to further evaluate the total B cell population (Figure 3A). This revealed high‐dimensional B cell subsets including transitional, naïve, follicular, and mature B cells (Figure 3B). Compared with naïve B cells, the levels of transitional and memory B cells were substantially reduced in the ALD‐induced mouse group (Figure 3C). This indicated that these cell types play a major role in ALD‐induced liver fibrosis. Similarly, a comparison of total gene expression between the groups showed significant up‐ and downregulation in B cell subsets (Figure 3D). Furthermore, we performed DEG analysis with the control and ALD‐induced mouse groups in each B cell subset by comparing the log2fold change and *p* values. Among the B cell subsets, transitional, follicular, and mature B cells showed significant gene expression changes, whereas naïve B cells did not (Figures 3E–H). The results revealed that more than 12 genes were differentially expressed in transitional B cells: *NKG7*, *GZMA*, *TRBC1*, *NRGN*, *TXK*, *CCL5*, *CD9*, and *MALAT1* were substantially upregulated; and *PLAC8*, *TPR*, *GM43305*, and *ARPC2* were considerably downregulated (Figure 3E). In follicular B cells, *EMB*, *TRBC*, *IL7R*, *RPL10*, and *LEF1* were upregulated, and 12 genes were downregulated (Figure 3F). In mature B cells, *FGFR1*, *S100A4*, *S100A6*, and *PLAUR* were upregulated, and eight genes were downregulated (Figure 3G). However, no significant changes were found in genes from naïve B cells (Figure 3H). The DEGs from B cell subsets were subjected to network analysis to understand their connections and biological function (Figure 3I). The genes from transitional B cells (*NKG7*, *CCL5*, and *NRGH*), naïve B cells (*RPL17*, *RPL37*, and *RPL39*), and follicular B cells (*MS4A1*, *CD79B*, *CD74*, *SYK*, *EBF1*, *IL7R*, and *LEF1*) were closely associated and shared a common biological role as receptor cell types.

Functional annotation of the expressed genes from subset populations of B cells was determined using Gene Ontology (GO) analysis. Functional annotation of transitional B cells revealed that several genes, including *PLAC8*, *ARPC2*, *CCL5*, *TXK*, *GZMA*, *TRBC1*, *TPR*, *NKG7*, *CD9*, *MALAT1*, and *NRGN*, are involved in the regulation of biological processes. Genes such as *PLAC8*, *ARPC2*, *CCL5*, *GZMA*, *TRBC1*, *TPR*, *CD9*, *MALAT1*, and *NRGN* are mostly engaged in protein complex binding, and *ARPC2*, *MALAT1*, and *NRGN* are involved in cellular communication (Figure 3J and Table S1). In follicular B cells, biochemical process genes such as *CD79B*, *IGHM*, *CD74*, *MEF2C*, *SYK*, *IGKC*, *TRBC1*, *LEF1*, *IL7R*, *MS4A1*, and *SIGLECG* are involved in B cell activation and cell receptor signaling pathways. Cellular component genes such as *CD79B*, *IGHM*, and *SYK* are involved in the B cell receptor complex, and molecular function genes such as *IGHM*, *IGKC*, and *TRBC1* are associated with immunoglobulin receptor binding (Figure 3K and Table S2). Mature B cells show similar results, while transitional B cells are involved in the regulation of biological processes (*CD83*, *LMO4*, *PLAUR*, *ATP1B1*, *H2‐AA*, *COQ7*, *AKAP12*, *VPREB3*, *TIFA*, *IGLC1*, *S100A4*, *SOX4*, *ATF3*, and *FGFR1*). Moreover, some cellular component genes (*LMO4*, *PLAUR*, *IGLC1*, *ATP1B1*, *H2‐AA*, *SOX4*, *ATF3*, and *FGFR1*) are involved in macromolecular complexes, and molecular function genes (*S100A6*, *ATP1B1*, *ATF3*, and *FGFR1*) are involved in protein binding (Figure 3L and Table S3). Finally, DEGs in naïve B cells such as *CD74*, *GDA*, *MT‐CO2*, *RPL37*, *RPL39*, and *RPL17* are involved in biochemical processes (cellular metabolic process) and molecular function (cytosolic ribosomal process), whereas *RPL37*, *RPL39*, and *RPL17* are involved in molecular function, especially ribosomal structure (Figure 3M and Table S4).

To determine the association between B cells and ALD, we conducted an in‐depth analysis of pro‐B cells by PG analysis. Pro‐B cells are precursor immature B cells, and types include progenitor B cells, pre–pro‐B cells, and pre‐B cells (Figure S3A). The population differences of each subset of pro‐B cells showed considerable changes. In particular, levels of progenitor and pre–pro B cells increased in the ALD‐induced mouse group (Figure S3B). Furthermore, network analysis of progenitor B cells revealed common functionality genes such as *S100A8* and *S100A9* and involvement of mostly positive inflammatory responses (Figure S3C,D). Additionally, genes such as *MT‐ATP6*, *MT‐CO2*, *MT‐CO3*, *MT‐ND1*, *S100A8*, and *S100A9* from pre–pro B and pre‐B cells were mostly involved in positive inflammatory responses, mitochondrial‐mediated function, and antioxidant pathways (Figure S3F,G and Table S5).

### 2.5 Eosinophils were Predominately Increased in the ALD Group

**FIGURE 4:**
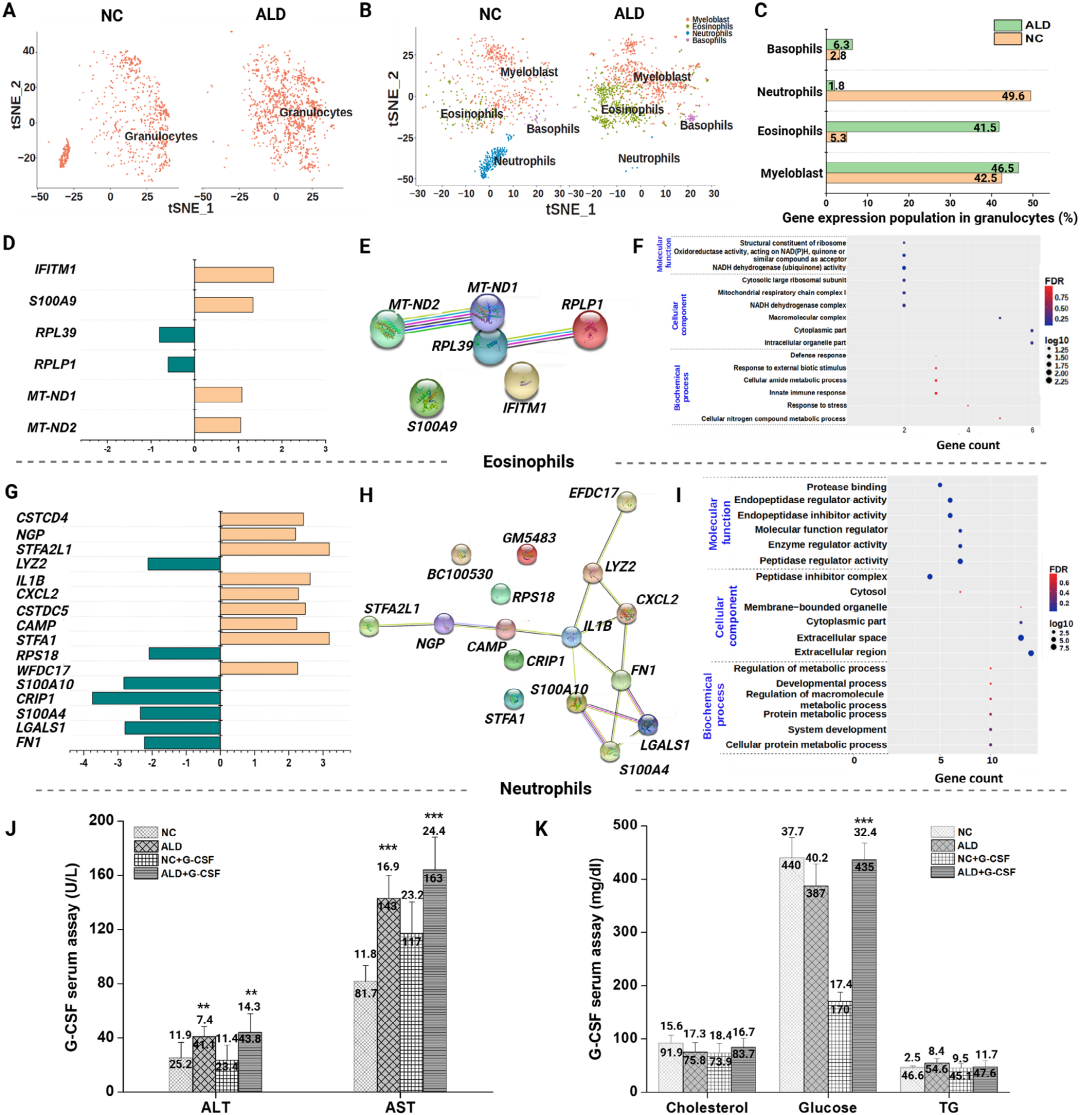
Immune profiling of granulocytes. (A) tSNE visualization of total granulocytes from NC and ALD‐induced mice groups. (B) Visualization of granulocyte subsets by applying the PhenoGraph clustering algorithm. (C) Population differences of each granulocyte subset from NC and ALD‐induced mice groups. (D–F) DEGs are depicted as a bar graph representing up‐ or downregulated genes, network analysis, and GO of eosinophils. (G–I) Bar diagram of DEGs, network analysis, and GO of neutrophils. GO enrichment analysis of significant eosinophils and neutrophils showing gene count with false discovery rate (FDR), log10, and *p* value. Statistically significant differences at 0.01 < *p* < 0.05, 0.001 < *p* < 0.01, and *p* < 0.001 are annotated as *, **, and ***, respectively. (J) Analysis of liver enzymes, such as ALT and AST, and (K) liver metabolism after in vivo administration of G‐CSF in both ALD and NC mice.

Granulocytes are an important biomarker of inflammatory responses in the body. To visualize the granulocyte subsets, we performed PG clustering of the total granulocyte population (Figure 4A). The granulocyte subsets identified showed four immune cell types of immature (myeloblast) and mature granulocytes of eosinophils, neutrophils, and basophils (Figure 4B). However, population characteristics were distinct among subsets (Figure 4C). The overall population of eosinophils was quite high in the ALD group, whereas that of neutrophils declined compared with those in the NC group. This indicates an imbalanced immune response that triggers an allergic reaction and destroys the immune defense system. Overall, the granulocyte gene expression profile was identified through log2fold change and *p* values, and an analysis of DEGs was conducted and displayed (Figure 4). Substantial gene expression from eosinophils and neutrophils was observed. Eosinophil genes such as *IFITM1*, *S100A9*, *MT‐ND1*, and *MT‐NTD2* were upregulated, whereas *RPLP1* and *RPL39* were downregulated (Figure 4D). Network analysis revealed common functionality of *MT‐ND1* and *MT‐ND2* genes from eosinophils (Figure 4E), and functional annotation of GO enrichment revealed a mitochondrial‐mediated respiratory chain complex via oxidoreductase activity by NADH dehydrogenase (Figure 4F). Other genes involved in the innate immune response by ribosomal protein synthesis, such as *IFTTM1*, *S100A9*, *RPL39*, and *RPLP1*, help in defense mechanisms (Figure 4F and Table S6). Furthermore, several genes in neutrophils were significantly downregulated, including *FN1*, *LGALS1*, *S100A4*, *CRIP1*, *S100A10*, *RPS18*, and *LYZ2*, whereas genes such as *WFDC17*, *STFA1*, *CAMP*, *CXCL2*, *IL1B*, *STFA2L1*, *NGP*, and *CSTDC4* were significantly upregulated (Figure 4G). Neutrophil genes that share a common functionality in network analysis include *LYZ2*, *FN1*, *CXCL2*, *STFA2L1*, *LGALS1*, *NGP*, *IL1B*, *S100A4*, *WFDC17*, *CAMP*, and *S100A10* (Figure 4H). GO enrichment analysis of expressed genes in neutrophils included cellular metabolic processes, extracellular regions, and activation of enzyme regulation processes (Figure 4I and Table S7).

Furthermore, scRNAseq analysis confirmed that granulocyte subsets play a major role in liver fibrosis through overexpression. To confirm these findings, we performed a validation study by in vivo administration of the commercially available drug Leucostim (a human recombinant granulocyte colony‐stimulating factor [G‐CSF]) for 17 days. The amount of liver enzymes including ALT and AST was measured, and a significant increment was confirmed in ALD‐induced mice groups compared with the NC group with or without G‐CSF (Figure 4J). Similarly, liver metabolism markers such as cholesterol, glucose, and triglyceride (TG) were also assessed, and their levels were compared between the ALD and NC groups (Figure 4K).

### 2.6 Increase in Classical and Decrease in Nonclassical Monocytes

**FIGURE 5:**
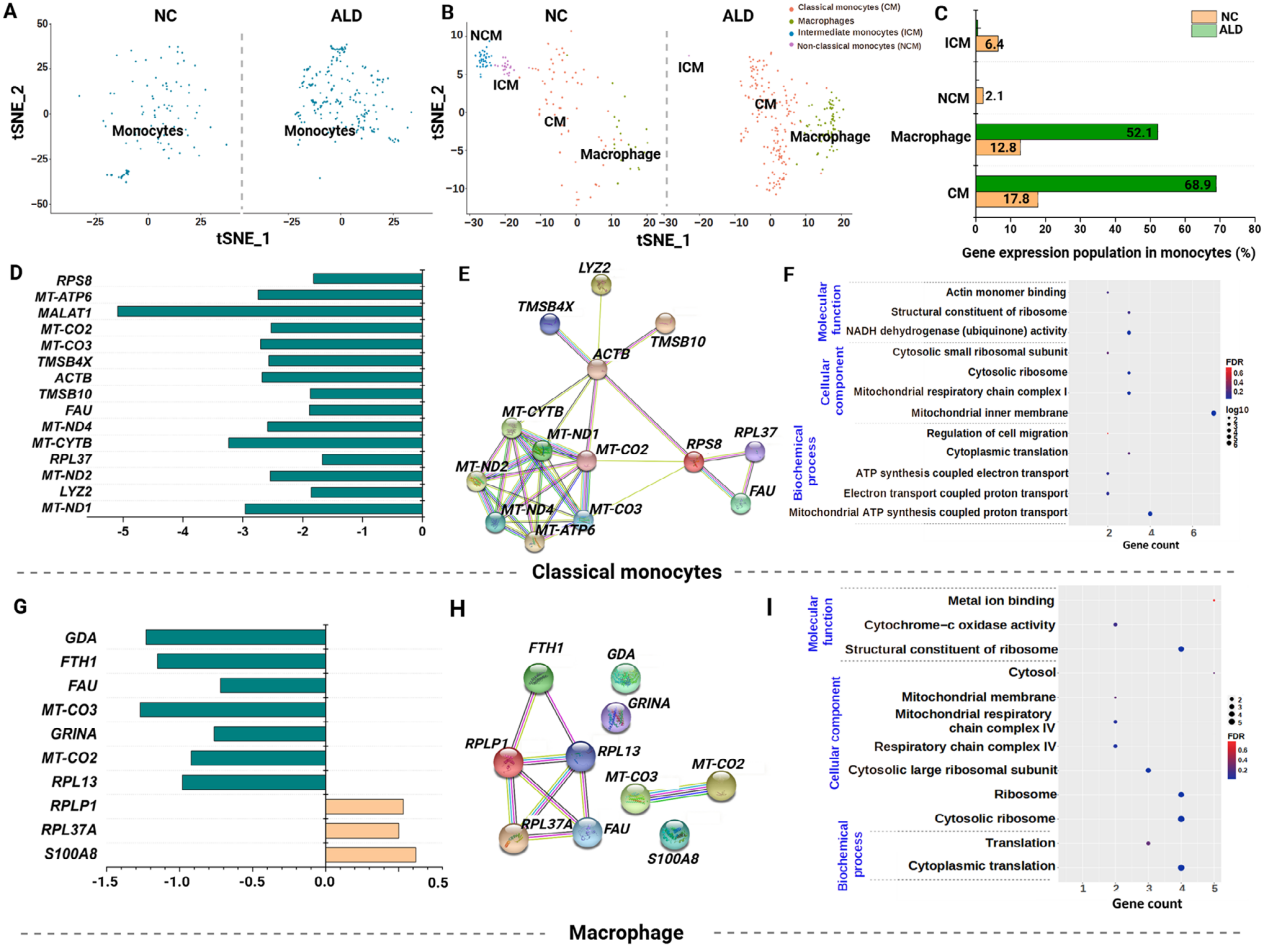
Deep immunological profiling of monocytes. (A) tSNE visualization of total monocytes from NC and ALD‐induced mice. (B) Monocyte subset visualization by the PhenoGraph clustering algorithm. (C) Population differences of monocyte subsets from NC and ALD‐induced mice. (D–F) The DEG expression, network profiling analysis, and GO enrichment analysis of classical monocytes. (G–I) The up‐ and downregulation of macrophage genes depicted as a bar graph, network profiling analysis, and GO enrichment analysis. GO enrichment analysis of significant monocyte subsets clusters showing gene count with FDR and log10, *p* value. The statistically significant differences at 0.01 < *p* < 0.05, 0.001 < *p* < 0.01, and *p* < 0.001 are annotated as *, **, and ***, respectively.

Monocyte populations from both NC and ALD‐induced mouse groups (Figure 5A) were further analyzed with the PG clustering algorithm into monocyte subsets. Reference marker gene expression analysis arranged the PG clustered monocyte subsets into classical, nonclassical, and intermediate monocytes and macrophages (Figure 5B).

The differences in the populations of monocyte subsets were significant. In particular, classical monocyte and macrophage populations were substantially increased in the ALD‐induced mouse group compared with the NC group (Figure 5C). In contrast, the nonclassical and intermediate monocyte populations were considerably reduced in the ALD‐induced mice compared with the NC group (Figure 5C). To conduct in‐depth profiling, we sequentially performed DEG analysis for each monocyte subset by comparing log2fold change and *p* values. DEGs from each subset were normalized to the NC group. Most of the genes from classical monocytes such as *MALAT1*, *MT‐ATP6*, *MT‐ND‐1*, *MT‐ND2*, *MT‐CYTB*, *MT‐CO2*, and *MT‐CO3* were significantly downregulated (Figure 5D). The network signaling DEGs from classical monocytes revealed cellular association and shared common functionality (Figure 5E). Genes such as *MT‐ATP6*, *MT‐ND‐1*, *MT‐ND2*, *MT‐CYTB*, *MT‐CO2*, and *MT‐CO3* from classical monocytes shared common functionality and were actively involved in mitochondrial‐mediated electron transport mechanism, whereas genes such as *TMSB4X* and *TMSB10* were actively involved in cellular migration (Figure 5F and Table S8). Furthermore, genes such as *RPS8* and *RPL37* from classical monocytes contributed to cytoplasmic ribosomes involved in protein translation (Figure 5F and Table S8).

Similarly, some of the genes from macrophages, such as *MT‐CO2*, *MT‐CO3*, *RPL13*, *GDA*, *FTH1*, and *FAU*, were considerably downregulated; however, a few genes, including *S100A8*, *RPL37A*, and *RPLP1*, was slightly upregulated (Figure 5G). Network signaling showed that ribosomal proteins such as *RPL37A*, *RPLP1*, *RPL13*, and *FTH1* shared common functionality. Similarly, the mitochondrial‐mediated electron transport genes *MT‐CO2* and *MT‐CO3* showed common functionality (Figure 5H). The GO analysis revealed similar pathways with classical monocytes, involving mitochondrial‐mediated electron transport as well as cytoplasmic ribosomal contributions (Figure 5I and Table S9).

For T cells, our study revealed that frequent alcohol consumption causes oxidative stress via an inflammatory response in CD4+T cells, indicating positive regulation of disease‐associated protein metabolism (Figure S4 and Tables S10–S12).

### 2.7 Comparative Analysis of DEGs in ALD and NC Groups

**FIGURE 6:**
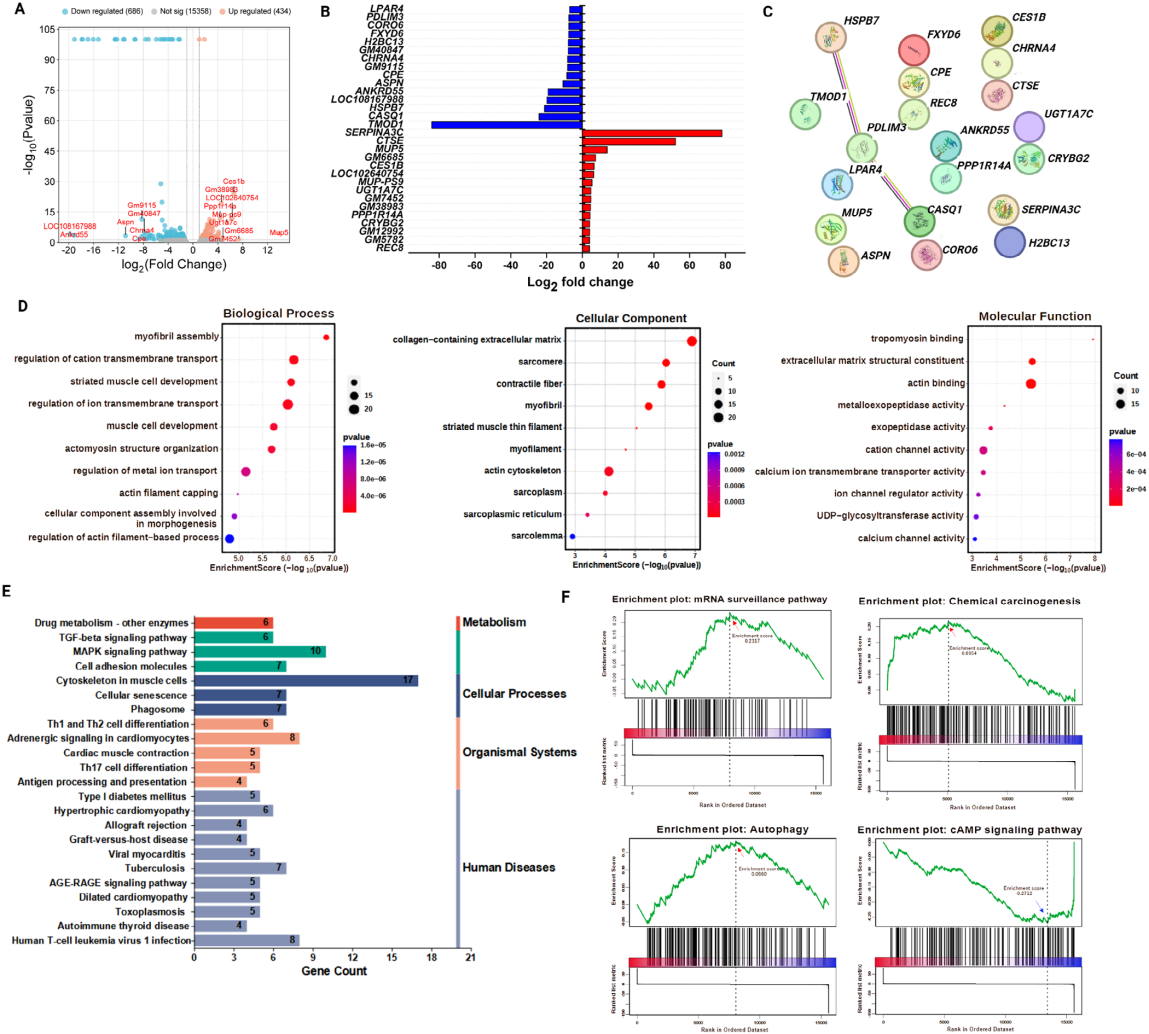
Bulk‐RNAseq analysis of liver tissue. (A) Volcano plot showing both up‐ and downregulated DEGs with log_2_ fold change values. (B) The most substantial DEGs are listed in a bar graph with their expression. (C) Network analysis was performed and the results were visualized for significant DEGs. (D) The GO enrichment analysis showed functional differences in DEGs. (E) Proposed molecular pathways of DEGs. (F) GSEA analysis of DEGs.

A bulk‐RNAseq analysis was conducted, and the data from ALD‐induced and NC mice groups were compared. A DEG analysis was performed and confirmed that more than 1120 of 16478 genes were expressed significantly and included both up‐ (434) and downregulated genes (686). The volcano plot displays these genes: *TMOD1*, *CASQ1*, *HSPB7*, *ANKRD55*, *ASPN*, *CPE*, *GM9115*, *CHRNA4*, and so on were downregulated substantially, whereas *SERPINA3C*, *CTSE*, *MUP5*, *GM6685*, *CES1B*, *MUP‐PS9*, *UGT1A7C*, and *PPP1R14A* were significantly upregulated (Figure 6A). The most significant genes are listed and visualized in a bar graph with their log2fold change values in Figure 6B. Similarly, significant genes were visualized through network analysis to explore the functional interactions between them. Among these, *HSPB7*, *LPAR4*, and *CASQ1* showed more numerous significant interactions than others (Figure 6C). The functional annotations of DEGs were determined through GO analysis and included biological processes, cellular components, and molecular functions as shown in Figure 6D. In biological processes, the regulation of metal ion transport was mainly upregulated, and collagen‐containing extracellular matrix was activated in cellular components (Figure 6D). The proposed pathways were listed based on various parameters including metabolism, cellular processes, organismal systems, and human diseases (Figure 6E). Additionally, gene set enrichment analysis (GSEA) performed on all the DEGs demonstrated that significantly upregulated pathways included autophagy, chemical carcinogenesis, and mRNA surveillance (Figure 6F).

## 3 Discussion

Although several studies have performed bulk‐level transcriptomic analysis of DEGs associated with ALD‐induced liver fibrosis [26, 27], cell‐specific target molecular pathways and genes involved in ALD have received less attention. The profiling of heterogeneous immune leukocytes and their subsets associated with ALD at the single‐cell level is more challenging. Therefore, in this study, we used a high‐dimensional scRNAseq analytical approach to evaluate the heterogeneity in the complex immune system and related single‐cell level expression that contributes to liver fibrosis. In addition to scRNAseq analysis, we performed bulk‐RNAseq analysis on liver tissues from ALD‐induced and control mice to develop a multimodal approach to alcohol‐induced liver fibrosis.

The liver fibrosis effect in ALD‐induced mice was validated by a histochemical comparison with healthy controls. The histochemical scoring of steatosis, NASH activity, and ballooning degeneration indicated fibrotic liver tissue with prolonged exposure to an alcoholic diet. Acute or persistent immunological responses from long‐term alcohol exposure cause irreversible liver damage [28, 29]. Our study demonstrated that the ballooning degeneration effect is a hallmark of NASH activity as well as disease progression [30], leading to cirrhosis and irreversible damage to the liver.

Furthermore, the biochemical analysis of parameters showed a significant elevation of liver enzymes such as ALT and AST in the ALD‐induced mice compared with the NC group. The elevation of ALT and AST indicates severe liver cell damage [31]. Moreover, prolonged alcohol exposure might increase the level of AST compared with that of ALT [32]. Our study confirmed a substantial increment in AST level compared with ALT, and liver metabolism was also slightly affected in the ALD‐induced mice compared with the control group. Overall, the in vivo evaluation in our study confirmed liver fibrosis in ALD‐induced mice.

Based on population changes and the expression of DEGs in major immune cell types, we analyzed and identified subsets with their transcriptomic changes. Initially, we discovered two distinct subsets of B cells, immature pro‐B cells and B cells, identified by reference marker genes. Since B cell heterogeneity may differ and play a critical role in liver fibrosis due to distinct functional characteristics, we performed in‐depth scRNAseq analysis to investigate B cell heterogeneity. The toxicity of alcoholic exposure resulted in apoptotic‐mediated cell death in circulating B cell populations [33] and resulted in population destruction in ALD‐induced mice. Subset identification of B cells by functional reference marker genes indicated that transitional B cells showed a significant population reduction in response to liver fibrosis. This may be associated with pathogenic progression of the upregulating gene *MALAT1*, as previously reported [34]. In addition, upregulated genes from transitional B cells, such as *TXK*, *TRBC1*, *GZMZ*, and *NKG7*, may cause high‐level liver injury following excess exposure to alcohol [35, 36, 37].

The innate immune response can be activated by increasing B cell maturation [35, 38]. Our study, in agreement with previous reports, found that the upregulation of *NRGN*, *CCL5*, and *CD9* genes may actively involve B cell maturation. However, population reduction in transitional B cells indicated inhibition of B cell maturation via downregulation of *ARPC2*, *PLAC8*, and *TPR* genes [35, 38]. Our results were in line with this finding. The population of mature B cells decreased significantly by downregulating genes that cause toxic effects upon alcohol consumption. These downregulated genes may be responsible for an imbalance in B cell function in response to pathogenic infection. The upregulation of *PLAUR*, *S100A4*, *S100A6*, and *FGFR1* in mature B cells was involved in disease‐associated cellular migration and tissue invasion, which may influence hepatocellular carcinoma with persistent liver injury [39, 40]. Similarly, due to the toxic effects of alcohol, most genes from follicular B cells were significantly downregulated, which may result in failure to activate the adaptive immune response by interacting with antigens from the periphery and secondary lymphoid organs to differentiate into plasma cells [41]. Finally, the cellular expression of genes such as *S100A8*, *S100A9*, *MT‐ND1*, *MT‐CO2*, *MT‐CO3*, and *MT‐ATP6* from pro B cell subsets demonstrated inflammation‐induced liver injury progression, involving mitochondrial oxidative phosphorylation‐induced energy loss [42, 43]. The population of pro‐B cell subsets, progenitor, pre–pro, and pre‐B cells showed significant collagen deposition via cellular differentiation from hepatic stellate cells into myofibroblasts, which caused liver injury in ALD‐induced mice [44].

Functional characteristics of granulocytes revealed differences in subsets including immature granulocytes (myeloblast), eosinophils, neutrophils, and basophils in the ALD‐induced mouse group. The granulocyte subset and neutrophils were considerably destroyed in the ALD‐induced mice, suggesting that prolonged alcohol consumption could deplete neutrophils and impair their patrolling functions [45]. The elevation of *STFA1* and *CXCL2* in the ALD‐induced group can lead to hyper‐inflammatory responses via activated neutrophil infiltration (Girbl et al. [67]; Patra et al. [63]) due to an enhanced inflammatory response to maintain a high alert defense mechanism at the injured site [46, 47]. Our findings also revealed overexpression of the neutrophil granule protein (NGP) and CAMP genes, which may actively contribute to neutrophil infiltration at the damaged region (Veglia et al. [64]). Moreover, this initiates dysfunction in patrolling neutrophils and causes further pathological conditions, such as hepatocellular carcinoma, in ALD‐induced mice by downregulating *RPS18* [48]. In addition to liver injury, this also promotes cirrhotic metastasis by upregulating the *CSTDC4* and *CSTDC5* genes [49]. Furthermore, immune system dysfunction caused by inhibition of neutrophil infiltration or macrophage‐mediated inflammatory response can lead to microbial infection (Bagheri‐Hosseinabadi et al. [65]; Ragland et al. [66]), as evidenced by downregulation of the immune defense‐associated lysozyme gene *LYZ2* and the inflammatory protein genes *S100A4* and *S100A10*. Other granulocyte subsets, such as eosinophils, were significantly increased in ALD‐induced mice compared with the NC group, suggesting that a persistent inflammatory response may promote pathological conditions in ALD [50]. Furthermore, the cellular expression of DEGs from eosinophils such as *IFITM1* and *S100A9* was associated with the progression of inflammatory‐mediated disease‐driven hepatocellular carcinoma [51] via upregulated mitochondrial oxidative phosphorylation genes (*MT‐ND1* and *MT‐ND2*) [43].

The granulocyte‐induced liver fibrotic effect was assessed in blood samples collected from mice with or without G‐CSF treatment. The ALD+G‐CSF‐treated group showed increases of both liver enzymes (ALT and AST) compared with the NC+G‐CSF group, which confirmed liver cell damage or fibrosis. Additionally, dysfunction of liver metabolism caused by alcoholic diets was noted, with an imbalance of free glucose in blood samples from ALD+G‐CSF and NC+G‐CSF. Overall, impaired granulopoiesis and granulocytopenia by chronic alcoholic exposure may influence the hemopoiesis of proinflammatory cytokines, which causes liver cell damage leading to fibrosis.

The differentiation of monocytes in complex cellular systems hinders determination of their role at the single‐cell level. Our scRNAseq analysis revealed monocyte heterogeneity, with subset populations identified as classical, nonclassical, and intermediate monocytes and differentiated macrophages based on the expression of reference marker genes. The expression of classical monocytes and macrophages in ALD‐induced mice was significantly higher than that in the NC group, suggesting that activation of proinflammatory cytokines may enhance liver injury by activated hepatic cells, resulting in alterations in the extracellular matrix [52]. Furthermore, repeated alcoholic consumption in the ALD‐induced mouse group resulted in immune system dysfunction due to the downregulation of genes from classical monocytes, which may fail to stimulate postinflammatory tissue repair and harmful ALD humoral immune response. The gene expression of macrophages was also downregulated in ALD‐induced mice, specifically *FTH1*, *MT‐CO2*, *MT‐C03*, *RPLP1*, and *RPL37A*, which induce mitochondrial‐mediated oxidative stress. In particular, *FTH1* expression modulates macrophage activation in response to immunological as well as microbial stimuli and is needed for cell protection against oxidative stress. Our study also revealed that frequent alcohol consumption causes oxidative stress via an inflammatory response in CD4+T cells, indicating positive regulation of disease‐associated protein metabolism (*RPL27A*, *RPLP2*, and *RPL13*), with induction of antioxidant activity (*S100A8* and *S100A9*) via proinflammatory immune cytokines [42]. Additionally, the toxic content of alcoholic byproducts from alcohol consumption in ALD‐induced mice was found to positively regulate an inflammatory cellular response (*ITGA4*, *CCR7*, *CD74*, *GZMA*, *ID2*, and *RORA*) in membrane‐bound organelles [53].

Most of the DEGs identified in this study were involved in liver fibrosis and dysfunction by changing the morphological features of the liver. Highly expressed genes, such as *SERPINA3C* and *MUP5*, are mostly involved in insulin‐like growth factor 1 signaling, which imbalances insulin levels and leads to related health issues [54]. Furthermore, elevation of the innate inflammatory response by upregulation of *CTSE* and *REC8* genes in ALD‐induced mice may cause a persistent hyper‐inflammatory response leading to damage of liver cells [55]. Additionally, genes such as *PPP1R14A* and *CRYBG2* were highly expressed and involved in the inhibition of protein phosphate and carbohydrate‐binding activity [56]. Similarly, the downregulated genes *TMOD1*, *ASPN*, *CASQ1*, and *PDLIM3* were mainly involved in muscle contraction. Cartilage formation in the liver may cause progressive liver fibrosis [57], and downregulation of the FXYD6 and CORO6 genes in ALD‐induced mice may result in progression of hepatocellular carcinoma [58, 59]. Overall, the bulk‐RNAseq analysis of liver tissues from the ALD‐induced mice confirmed the progression of liver fibrosis by transcriptomic changes in DEGs. To support this finding, GO studies revealed that the functional annotations of DEGs showed mostly liver tissue modification and fibrotic progression. Finally, selective pathways obtained from GSEA indicated that alcoholic exposure enhanced autophagy and chemical carcinogenesis, downregulating the cAMP signaling pathway, which is involved in vital functions such as metabolism, secretion, and gene transcription [60].

## 4 Conclusion

**FIGURE 7:**
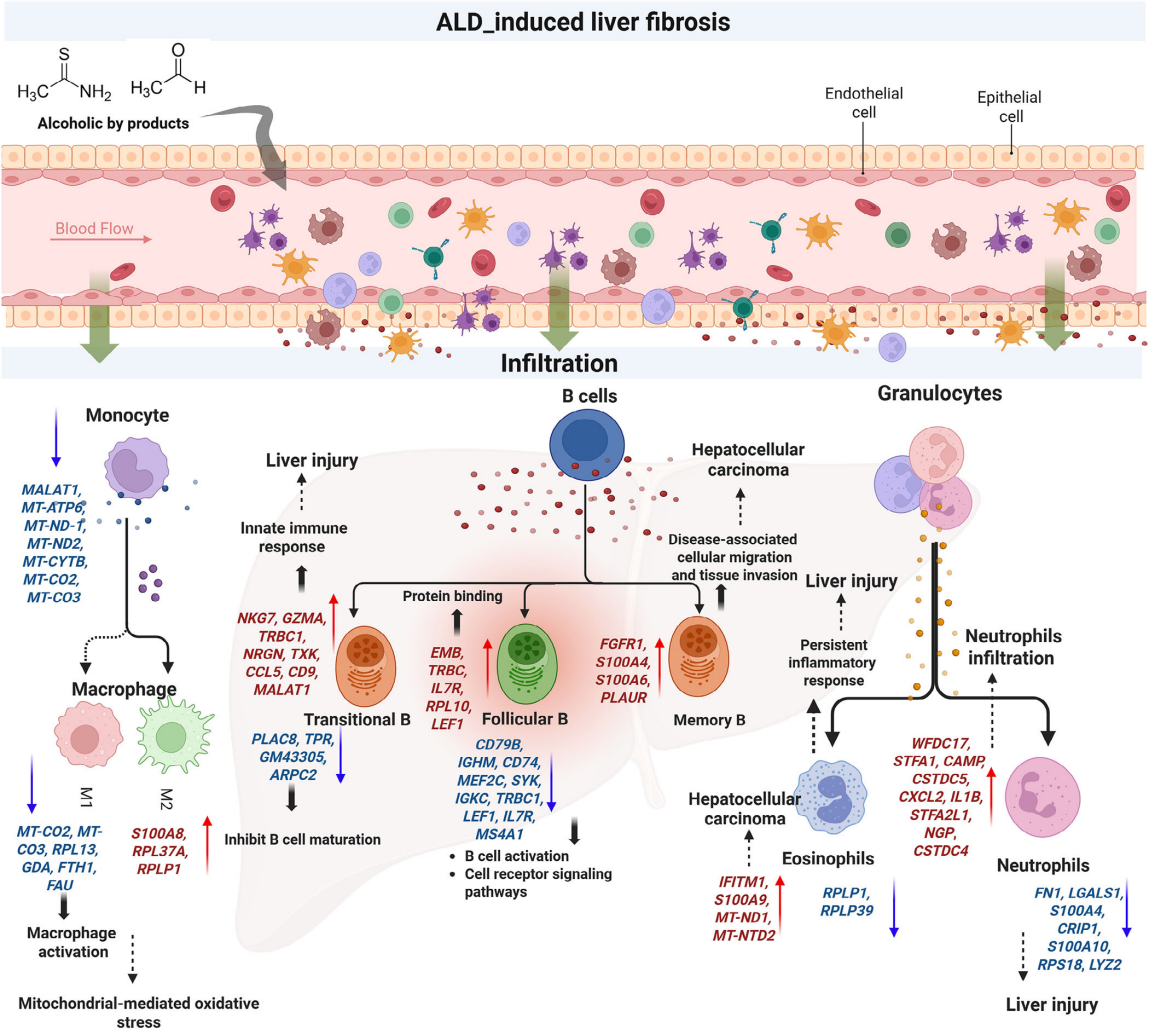
Proposed schematic representation of immune cell types and their DEGs involved in alcohol‐induced liver fibrosis.

Overall, our study used high‐dimensional scRNAseq analysis to discover that heterogeneous immune leukocytes from ALD‐induced and healthy control mice were accountable for ASH at the single‐cell level (Figure 7). Specifically, significant cell types (B cells, granulocytes, and monocytes) and their heterogeneous subsets involved in ASH were identified using automated singleR cell annotation with marker gene expression. A population decrement in B cells and their subsets (transitional and follicular B cells) denoted an important indication of alcohol‐induced liver fibrosis. In particular, up‐ and downregulated genes from B cell subsets initiated an innate proinflammatory response. Similarly, neutrophil deficiency in the ALD‐induced mice was another key finding in ASH. An increase in eosinophils diverts further complications in liver fibrosis and emphasizes the functional heterogeneity of granulocyte subsets. The alleviated monocytes in ALD‐induced mice increased activation of macrophage‐mediated inflammatory responses, which may lead to chronic liver inflammation. Additionally, bulk‐RNA sequencing on liver tissues from ALD‐induced mice confirmed liver cell damage, and muscle contraction may cause hepatocellular carcinoma progression. Though our pilot study demonstrates heterogeneous immunological responses from both mouse peripheral blood mononuclear cells (mPBMCs) and liver tissue of ALD‐induced mice, the limited sample size could not corroborate our findings with certainty. Furthermore, validation of the immunological responses of specific cell types, such as B cells, granulocytes, and monocytes, in the heterogeneous environment is warranted to confirm our findings.

In summary, our scRNAseq study identified potential ALD biomarkers to distinguish specific cell types and their subsets that improve drug selection and therapeutic efficacy. Second, our pilot study helps to design potential therapeutic targets or combinations of drugs to predict the specific cell types of immunological responses in a heterogeneous environment. Third, our current findings can be used to develop prospective treatment targets for various alcohol‐related diseases such as hepatitis B, fatty liver, and other immunodeficiency disorders that weaken the immune defense mechanism.

## 5 Materials and Methods

### 5.1 Experimental Mouse Group

Male C57BL/6N mice were purchased from Orient Bio (Seongnam‐si, Gyeonggi‐do, Republic of Korea) at the age of 6 weeks. The mice were house in a room with a 12 h light/dark cycle, a temperature‐regulated environment (23 ± 2°C), and specific pathogen‐free housing. The study adhered to the ethical guidelines set by the Institutional Animal Care and Use Committee at Hanyang University. Ethical clearance number HY‐IACUC‐20‐0043. The mice were divided into two groups, normal mice (NC; *n* = 5) and alcohol‐treated mice (AC; *n* = 5). The NC group was fed a normal diet for 22 days, and the AC group was fed a normal diet for 5 days and an alcohol‐treated diet for 17 days.

### 5.2 In Vivo Validation of Alcoholic Induction of Liver Fibrosis

The liver fibrosis effect was confirmed in the ALD‐induced mice group by histological assessment including H&E and Sirius staining. Similarly, blood samples from each group were used for various biochemical investigations, including ALT, AST, cholesterol, glucose, and TGs. Furthermore, to validate the role of granulocyte subsets in liver fibrosis, we exposed the mice to G‐CSF (Leucostim, 30 µg/kg; Dong‐A Pharmaceutical Co., Ltd, Seoul, Republic of Korea) as previously described by Nam et al. [61]. After 17 days, the animals were sacrificed, and blood and liver samples were collected for further examination.

### 5.3 Experimental Analysis of scRNAseq Analysis

**FIGURE 8:**
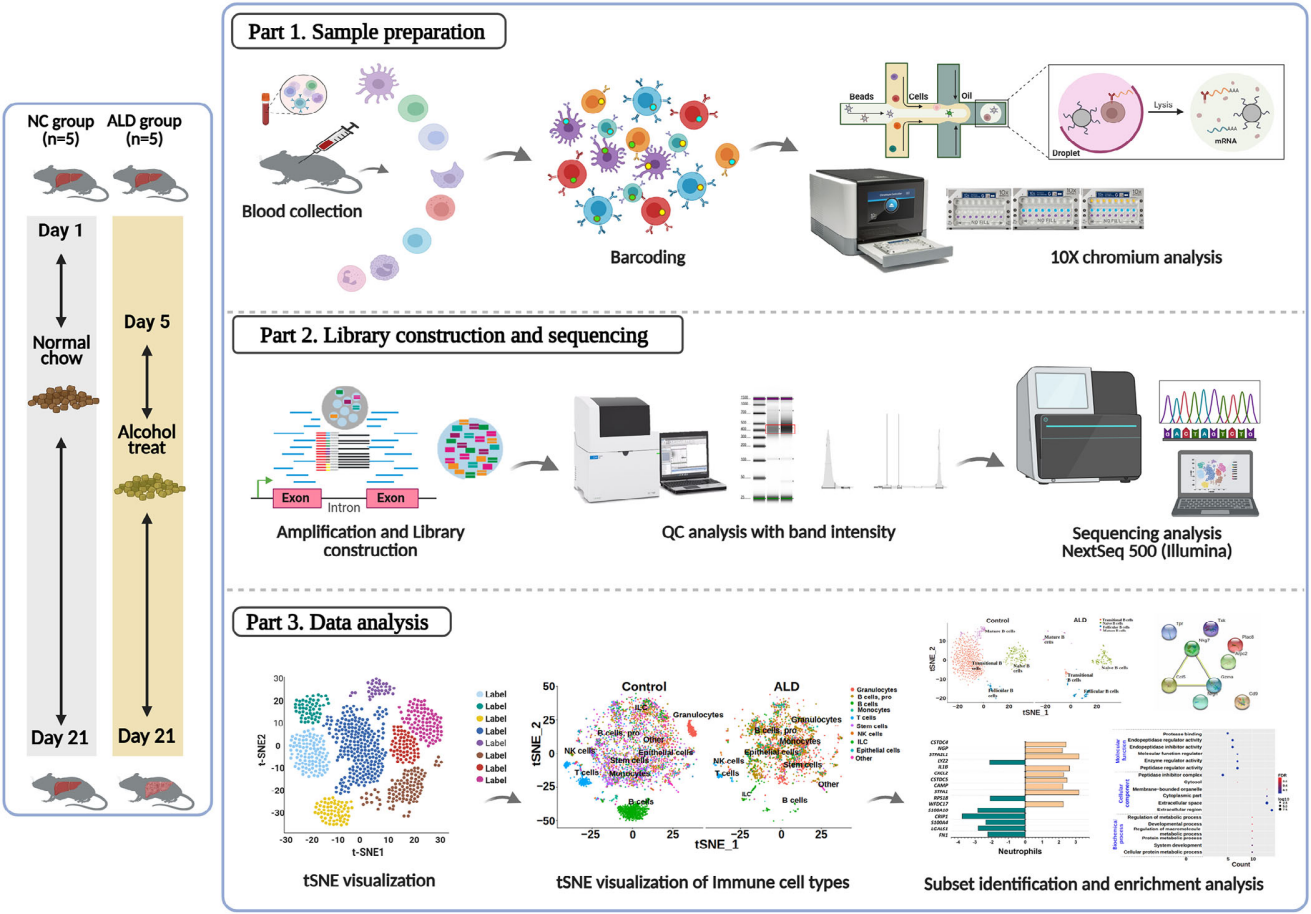
Scheme of scRNAseq analysis workflow. The treatment period of the ALD‐induced mice was 21 days. Blood samples for scRNAseq analysis were collected from both groups beginning on day 21. The 10× chromium analysis, library preparation, and sequencing were carried out at the single‐cell level to identify phenotypes and observe transcriptomes. Data visualization and processing were used to evaluate the immunological response of heterogeneous immune cell types.

mPBMCs were isolated from blood samples from each NC and ALD‐induced mouse group, pooled, and washed twice with cultured medium (RPMI‐1640) before analysis using a 10× chromium single‐cell analyzer (10× Genomics Chromium, Pleasanton, CA, USA). Approximately 17,000 cells were processed, and 10,000 cells per group were selected at the target cell recovery by the 10× controller. GEM generation and barcoding were conducted with single‐cell 3′ v3.1 gel beads, which are composed of a master mix with cell surface protein labels and partitioning oil added to a chromium chip (Figure 8). The cDNA amplification and post‐GEM‐RT cleanup steps were completed according to the manufacturer's instructions (Next GEM single‐cell kit V3.1, Pleasanton, CA, USA). A 3′ gene expression library was constructed with amplified cDNA using P5, P7, I7, and i5 sample indexes, and TruSeq read 2 was added via end repair, A‐tailing, and adaptor ligation as directed by the manufacturer's instructions (Figure 8). The library‐constructed samples were then sequenced using an Illumina NextSeq 500 sequencer to investigate the heterogeneous immune leukocyte populations from the control and ALD‐induced mouse models. A quality analysis was performed for all the samples using a tape station with D1000 and D5000 markers (Agilent Technologies).

### 5.4 Data Visualization of scRNAseq Analysis

The amplified sequences were used to construct a library using the chromium library protocol. NextSeq 500 (Illumina) was used for further sequencing to investigate the heterogeneous immune leukocyte populations from control and ALD‐induced mice models (Figure 1). We used t‐distributed stochastic neighbor embedding (t‐SNE) projection to visualize the high‐dimensional scRNAseq data and to observe the qualitative transcriptomic changes in the NC and ALD‐induced mouse groups. Each cell in the t‐SNE plot was assigned to a location on a 2D plot. The changes in locations of the dots demonstrated the influence of both the NC and ALD‐induced mouse groups. The “RunPCA” (npcs = 30) and “RunTSNE” (dims = 1:20) functions were applied to the integrated Seurat object. The “Dimplot” function created t‐SNE plots with cell types as labels. We generated an overall t‐SNE plot of all cells, with major types labeled, followed by a t‐SNE plot with subtype labels for each major cell type.

### 5.5 Bulk‐RNAseq Analysis

As previously described by Yuan et al. [62], liver tissue samples were dissected from the ALD and NC groups, and RNA was extracted using TissueLyzer (Qiagen, Hilden, Germany). The cDNA was prepared using 500 ng of isolated RNA and double‐stranded cDNA as previously reported [62]. The data were received and preprocessed to trim the reads using FAST‐0.23.4. STAR v2.7.1a was used to align reads, and HTSeq‐2.0.3 was used to obtain the raw read counts.

### 5.6 Functional Annotation of scRNAseq and Bulk‐RNAseq Analysis

To compare the transcriptomic profiles of control and ALD‐induced mice, we performed DEG analysis after cell annotation (SingleR) to determine which genes were upregulated or downregulated. The method for analyzing DESeq2 from different groups and comparing the functional differences used the “Findmarkers” tool in the R Seurat package. String network analysis (https://string‐db.org/) was used to investigate how the identified DEGs interact with each other or whether they share common functionality in NC and ALD‐induced mouse groups. GO analysis was performed using the DAVID platform (https://david.ncifcrf.gov/home.jsp) to reveal functional roles including biochemical processes, cellular components, and molecular functions. The basic tenet of ontology analysis is that the DAVID platform identifies the ontologies pertinent to each gene before identifying those involving several DEGs and summarizes these ontologies. We listed the gene ontologies in the order of importance based on the number of input genes included in each ontology. To evaluate the data in each group, we selected the top three to five ontologies for each category.

### 5.7 Statistical Analysis

Statistical significance was assessed using the Mann–Whitney *U* test, with *p* < 0.05 considered a significant difference.

## Author Contributions

THY and HP conceived the project. HP and SP performed experiments. HP and SP acquired and analyzed experimental data. SP, HP, JEK, KHY, and XX contributed to the interpretation of the experiments. HP wrote the original draft manuscript. HP and THY reviewed and edited the manuscript. THY and DWJ supervised the work. THY and DWJ acquired the funding. All authors contributed and approved the submitted version of the article.

## Ethics Statement

The study adhered to the ethical guidelines set by the Institutional Animal Care and Use Committee at Hanyang University. Ethical clearance number HY‐IACUC‐20‐0043.

## Conflicts of Interest

Tae‐Hyun Yoon is an employee of Yoon Idea Lab. Co. Ltd. but has no potential relevant financial or nonfinancial interests to disclose. The other authors declare no conflicts of interest.

## Supporting information




**Supporting Figure 1**: Quality check analysis. (A) Unique molecular identifiers (UMIs) were used to confirm the high quality of cells. (B) A UMI filter was used for gene counts. (C) The probability of doublet count was measured using a frequency spectrum. (D) QC of cDNA was analyzed by Tape Station with a D5000 marker. (E) QC on library construed samples was conducted by using Tape Station with a D1000 marker. (F) Concentration and base pair size of both samples.
Cell Ranger was used to process the fastq files of datasets for the different mouse groups. During the preprocessing steps, we excluded low‐quality genes by setting gene numbers <500 or >6000 throughout the preprocessing phases. Dead cells were eliminated by setting mitochondrial unique molecular identifiers (UMI) to more than 19% of transcripts (Figure 2). Doublets were identified and used to set the median threshold, assisting in the removal of low‐quality datasets from further data processing. After preprocessing, the total number of genes was identified after applying the UMI filter, and data from ALD 3683 cells and NC 5633 cells were aggregated using the function IntegrateData. The data were then integrated and input into the R Seurat package (4.0.6) for further analysis using R studio and a later version of the R programming language (R 3.3.0). From the barcodes, features, and matrix files, the “read10×” function created a Seurat object. An unsupervised automated clustering algorithm was used in conjunction with the SingleR packages for cell annotation. To identify immune cell types, we used the ImmGenData reference in SingleR [24].
**Supporting Figure 2**: Reference marker gene expression to identify the heterogeneous immune cell types by scRNAseq analysis.
**Supporting Figure 3**: Immune profiling of pro‐B cells. (A) tSNE visualization of pro‐B cells from NC and ALD‐induced mouse groups and visualized B‐cell subsets by applying the PhenoGraph clustering algorithm. (B) Population differences of each pro‐B cell subset from NC and ALD‐induce mouse groups. (C, D, and E) Network profiling analysis and (F and G) GO enrichment profiling of the pro‐B cells subsets with DEGs in NC and ALD‐induced mouse groups.
**Supporting Figure 4**: Deep immunological profiling of T‐cells. (A) tSNE visualization of total T‐cells from NC and ALD‐induced mouse groups. (B) Identification of T‐cell subsets by applying the PhenoGraph clustering algorithm. (C) Population differences of each T‐cell subset from NC and ALD‐induced mouse groups. (D, E, and F) The DEG expression, network profiling analysis, and enrichment analysis GO of naïve CD4+T. (G, H, and I) The DEG expression, network profiling analysis, and enrichment analysis GO of CD4+T helper cells. (J, K, and L) The DEG expression, network profiling analysis, and enrichment analysis GO of CD8+T killer cells.
**Supporting Table 1**: Gene ontology enrichment analysis of transitional B‐cells.
**Supporting Table 2**: Gene ontology enrichment analysis of follicular B‐cells.
**Supporting Table 3**: Gene ontology enrichment analysis of mature B‐cells.
**Supporting Table 4**: Gene ontology enrichment analysis of naïve B‐cells.
**Supporting Table 5**: Gene ontology enrichment analysis of pro B‐cells.
**Supporting Table 6**: Gene ontology enrichment analysis of eosinophils.
**Supporting Table 7**: Gene ontology enrichment analysis of neutrophils.
**Supporting Table 8**: Gene ontology enrichment analysis of classical monocytes.
**Supporting Table 9**: Gene ontology enrichment analysis of macrophages.
**Supporting Table 10**: Gene ontology enrichment analysis of naïve CD4+T‐cells.
**Supporting Table 11**: Gene ontology enrichment analysis of CD4+T helper cells.
**Supporting Table 12**: Gene ontology enrichment analysis of CD8+T killer cells.

## Data Availability

The mouse single‐cell RNA‐seq datasets generated in this study have been deposited in the NCBI repository (https://www.ncbi.nlm.nih.gov/sra/?term=PRJNA1241350), and the SRA accession numbers is PRJNA1241350 (SRR32869507).
