# Supplementary material for: Discovering Heterogeneous Leukocytes Subsets Associated With Alcoholic Steatohepatitis by scRNAseq Analysis
Source: MedComm (2020). 2025 Nov 2;6(11):e70448. doi: 10.1002/mco2.70448 (PMC12580407; doi:10.1002/mco2.70448)
Supplement: Supplementary file 1 — Supporting Figure 1: Quality check analysis. (A) Unique molecular identifiers (UMIs) were used to confirm the high quality of cells. (B) A UMI filter was used for gene counts. (C) The probability of doublet count was measured using a frequency spectrum. (D) QC of cDNA was analyzed by Tape Station with a D5000 marker. (E) QC on library construed samples was conducted by using Tape Station with a D1000 marker. (F) Concentration and base pair size of both samples. Cell Ranger was used to process the fastq files of datasets for the different mouse groups. During the preprocessing steps, we excluded low‐quality genes by setting gene numbers <500 or >6000 throughout the preprocessing phases. Dead cells were eliminated by setting mitochondrial unique molecular identifiers (UMI) to more than 19% of transcripts (Figure 2). Doublets were identified and used to set the median threshold, assisting in the removal of low‐quality datasets from further data processing. After preprocessing, the total number of genes was identified after applying the UMI filter, and data from ALD 3683 cells and NC 5633 cells were aggregated using the function IntegrateData. The data were then integrated and input into the R Seurat package (4.0.6) for further analysis using R studio and a later version of the R programming language (R 3.3.0). From the barcodes, features, and matrix files, the “read10×” function created a Seurat object. An unsupervised automated clustering algorithm was used in conjunction with the SingleR packages for cell annotation. To identify immune cell types, we used the ImmGenData reference in SingleR [24]. Supporting Figure 2: Reference marker gene expression to identify the heterogeneous immune cell types by scRNAseq analysis. Supporting Figure 3: Immune profiling of pro‐B cells. (A) tSNE visualization of pro‐B cells from NC and ALD‐induced mouse groups and visualized B‐cell subsets by applying the PhenoGraph clustering algorithm. (B) Population differences of each pro‐B cell [file MCO2-6-e70448-s001.docx]

**Discovering heterogeneous leukocytes and subsets associated with alcoholic steatohepatitis by scRNAseq analysis**

Haribalan Perumalsamy^1,2,3^, Sehee Park^3^, Ji Eun Kim^4^, Xiao Xiao^3^, Kim Hye Young^5^, Dae Won Jun^4,6^*, Tae-Hyun Yoon^1,3,7,8^*

^1^Research Institute for Convergence of Basic Science, Hanyang University, Seoul 04763, Republic of Korea

^2^Center for Creative Convergence Education, Hanyang University, Seoul 04763, Republic of Korea

^3^Department of Chemistry, College of Natural Sciences, Hanyang University, Seoul 04763, Republic of Korea

^4^Department of Translational Medicine, Graduate School of Biomedical Science and Engineering, Hanyang University, Seoul, Republic of Korea

^5^Department of Surgery, Institute of Surgery, Kangbuk Samsung Hospital, Sungkyunkwan University School of Medicine, Seoul, Republic of Korea

^6^ Department of Internal Medicine, College of Medicine, Hanyang University, Seoul, Republic of Korea

^7^Institute for Next-Generation Material Design, Hanyang University, Seoul 04763, Republic of Korea

^8^Yoon Idea Lab. Co. Ltd, Seoul 04763, Republic of Korea

*** Correspondence:
Prof. Dr. Dae Won Jun**,

Department of Internal Medicine,

College of Medicine, Hanyang University, Seoul, Korea

[noshin@hanyang.ac.kr](mailto:noshin@hanyang.ac.kr)

**Prof. Tae Hyun Yoon, Ph.D**

Department of Chemistry, College of Natural Sciences

Hanyang University, Seoul, 04763, Republic of Korea

Phone number: 82-2-2220-4593

Fax number: 82-2-2299-0762

[taeyoon@hanyang.ac.kr](mailto:taeyoon@hanyang.ac.kr)


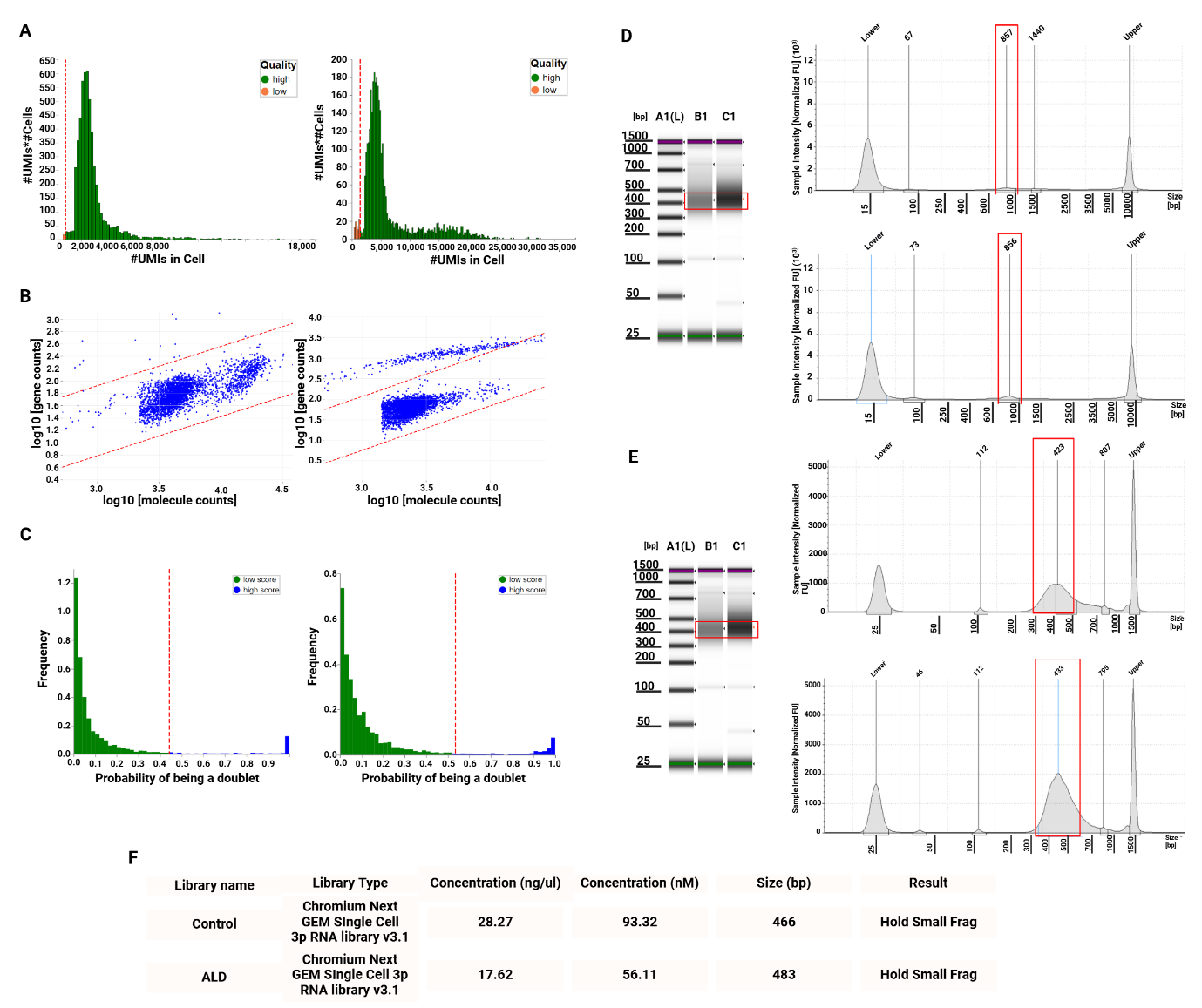


**Figure S1**. **Quality check analysis** (**A**) Unique molecular identifiers (UMIs) were used to confirm the high quality of cells. (**B**) A UMI filter was used for gene counts. (**C**) The probability of doublet count was measured using a frequency spectrum. (**D**) QC of cDNA was analyzed by Tape Station with a D5000 marker. (**E**) QC on library construed samples was conducted by using Tape Station with a D1000 marker (**F**) Concentration and base pair size of both samples.

Cell Ranger was used to process the fastq files of datasets for the different mouse groups. During the preprocessing steps, we excluded low-quality genes by setting gene numbers <500 or >6000 throughout the preprocessing phases. Dead cells were eliminated by setting mitochondrial unique molecular identifiers (UMI) to more than 19% of transcripts (**Figure 2**). Doublets were identified and used to set the median threshold, assisting in the removal of low-quality datasets from further data processing. After preprocessing, the total number of genes was identified after applying the UMI filter, and data from ALD 3683 cells and NC 5633 cells were aggregated using the function IntegrateData. The data was then integrated and input into the R Seurat package (4.0.6) for further analysis using R studio and a later version of the R programming language (R 3.3.0). From the barcodes, features, and matrix files, the "read10×" function created a Seurat object. An unsupervised automated clustering algorithm was used in conjunction with the SingleR packages for cell annotation. To identify immune cell types, we used the ImmGenData reference in SingleR [24].


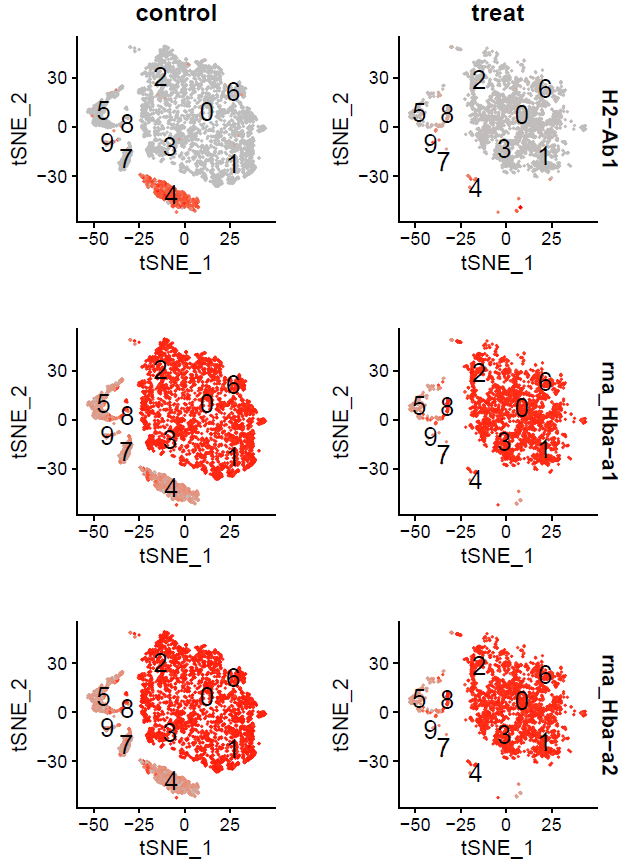

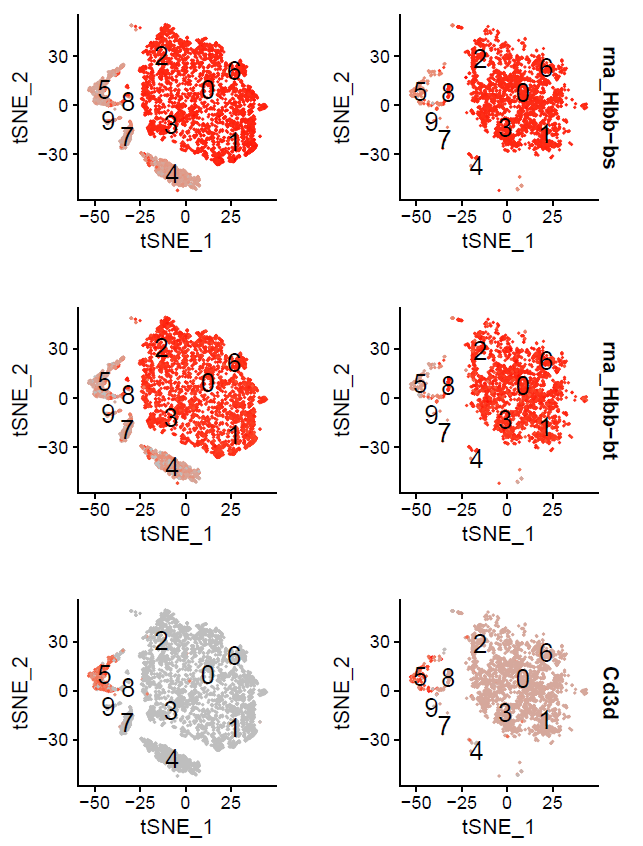

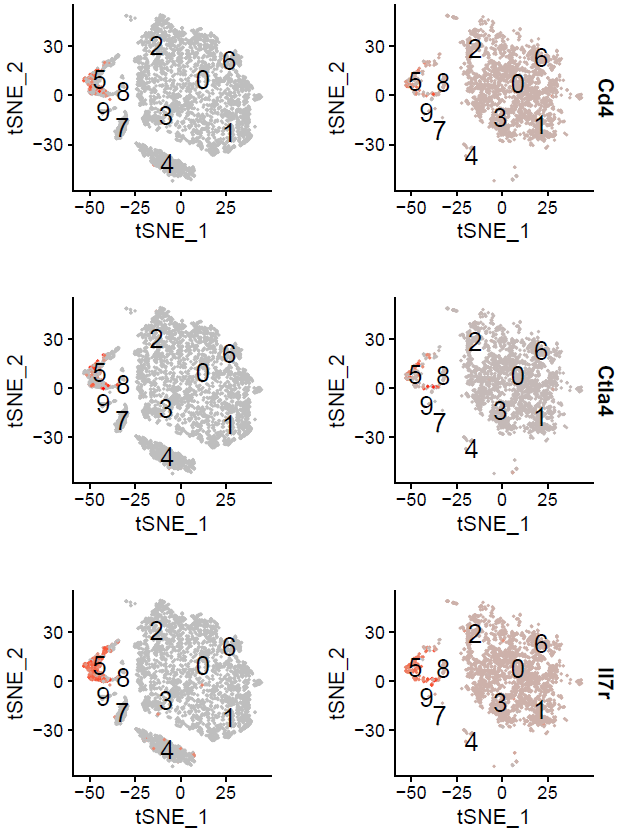


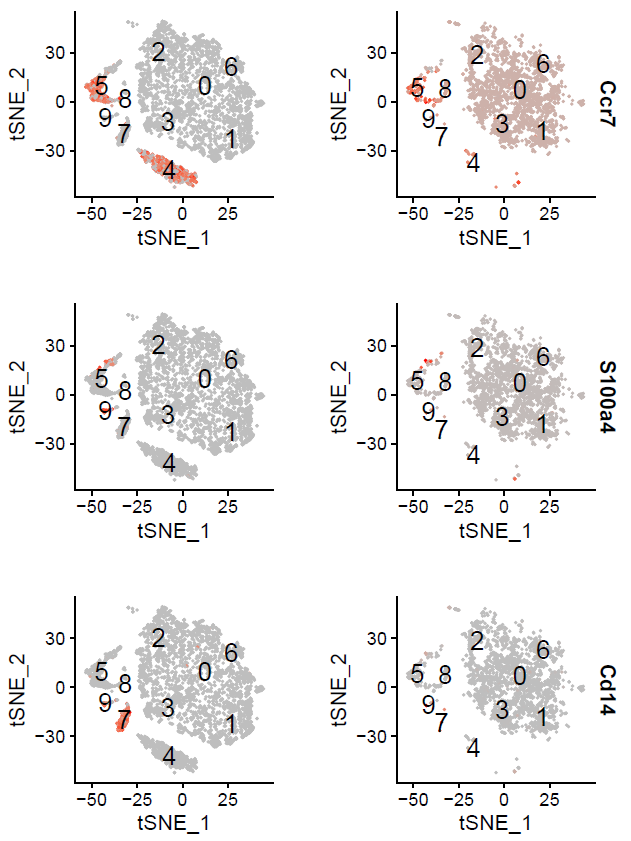

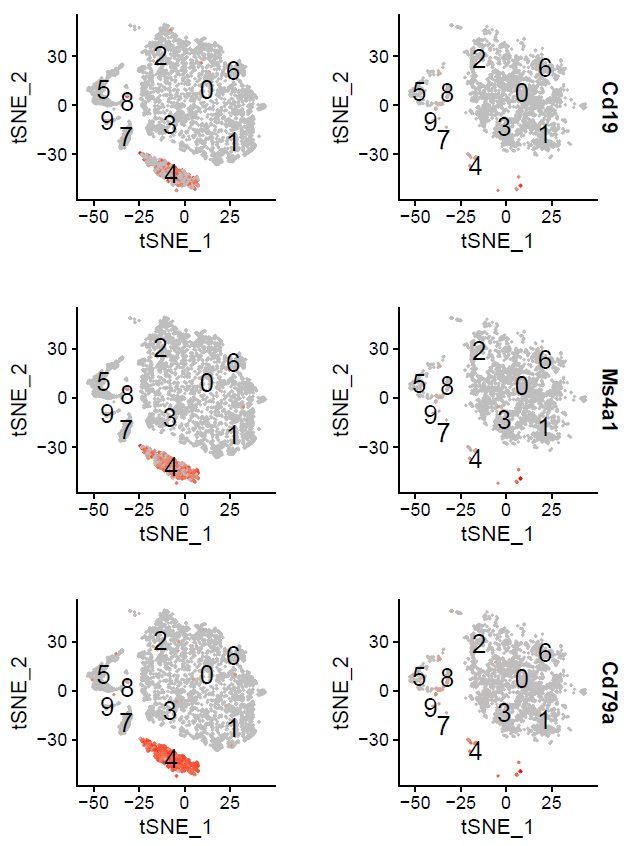

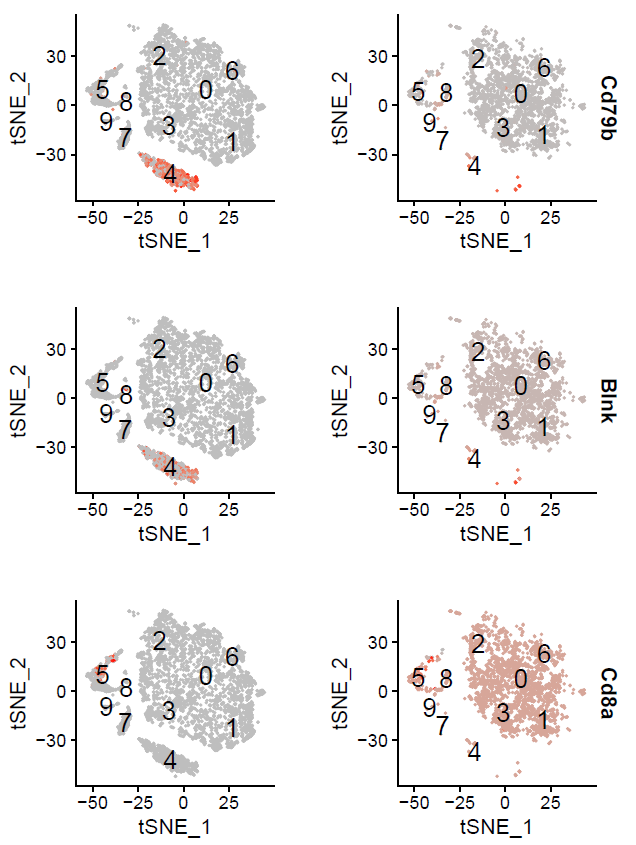


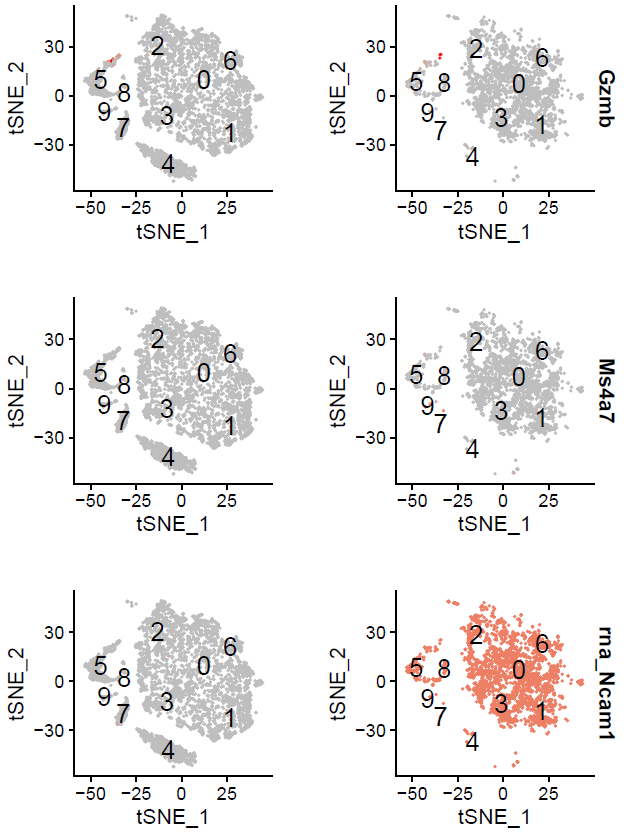

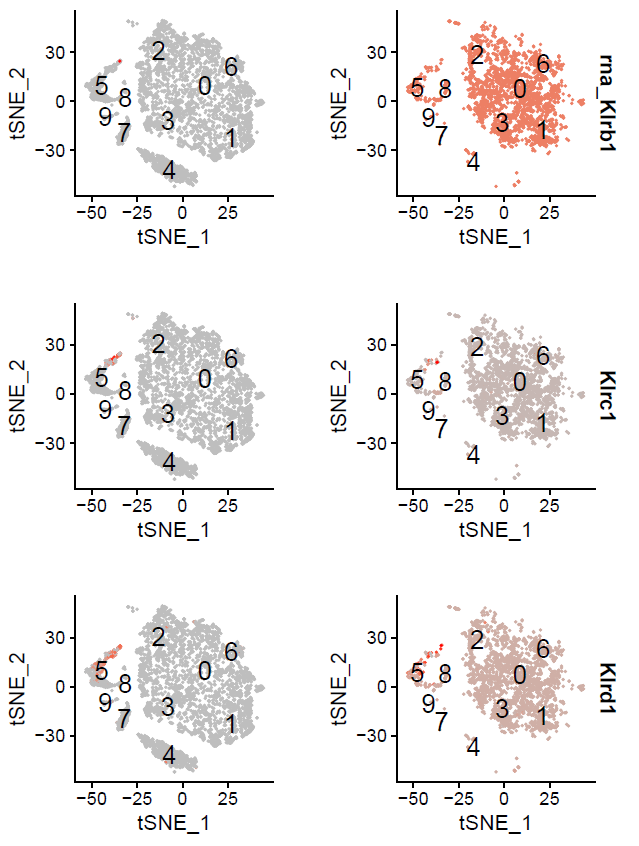

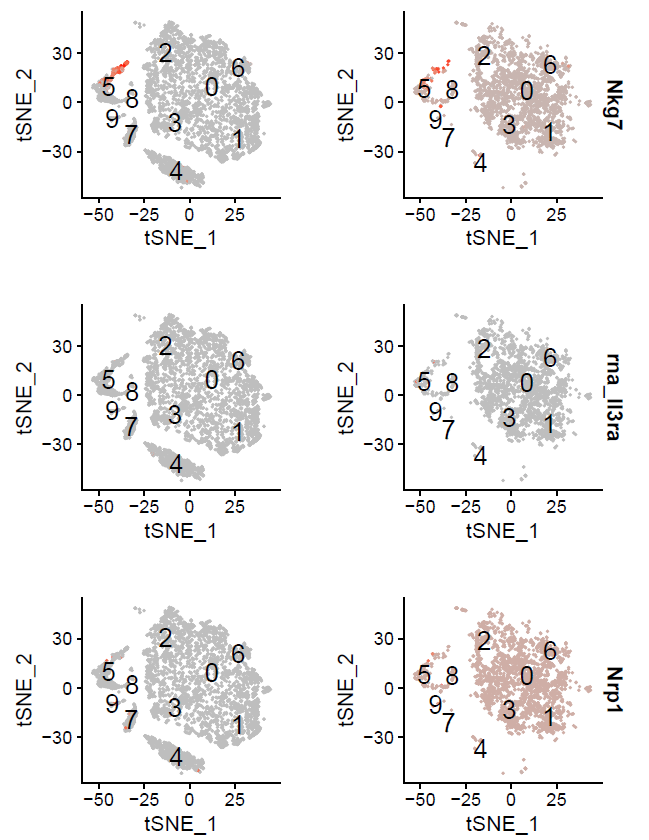


**Figure S2**. Reference marker gene expression to identify the heterogeneous immune cell types by scRNAseq analysis


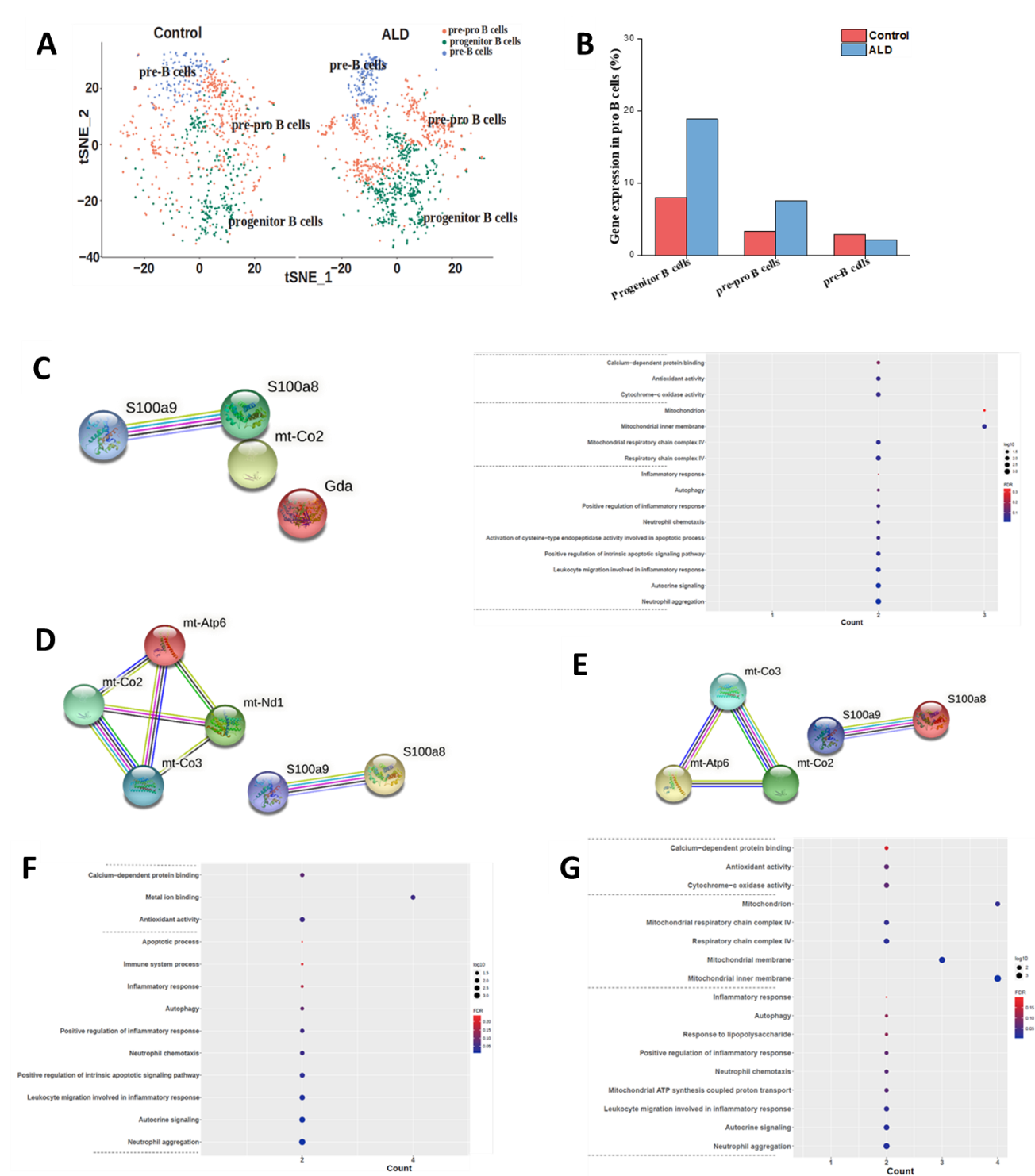


**Figure S3**. **Immune profiling of pro-B cells** (**A**) tSNE visualization of pro-B cells from NC and ALD-induced mouse groups and visualized B-cell subsets by applying the PhenoGraph clustering algorithm. (**B**) Population differences of each pro-B cell subset from NC and ALD-induce mouse groups. (**C, D, E**) Network profiling analysis and (**F, G**) GO enrichment profiling of the pro-B cells subsets with DEGs in NC and ALD-induced mouse groups.


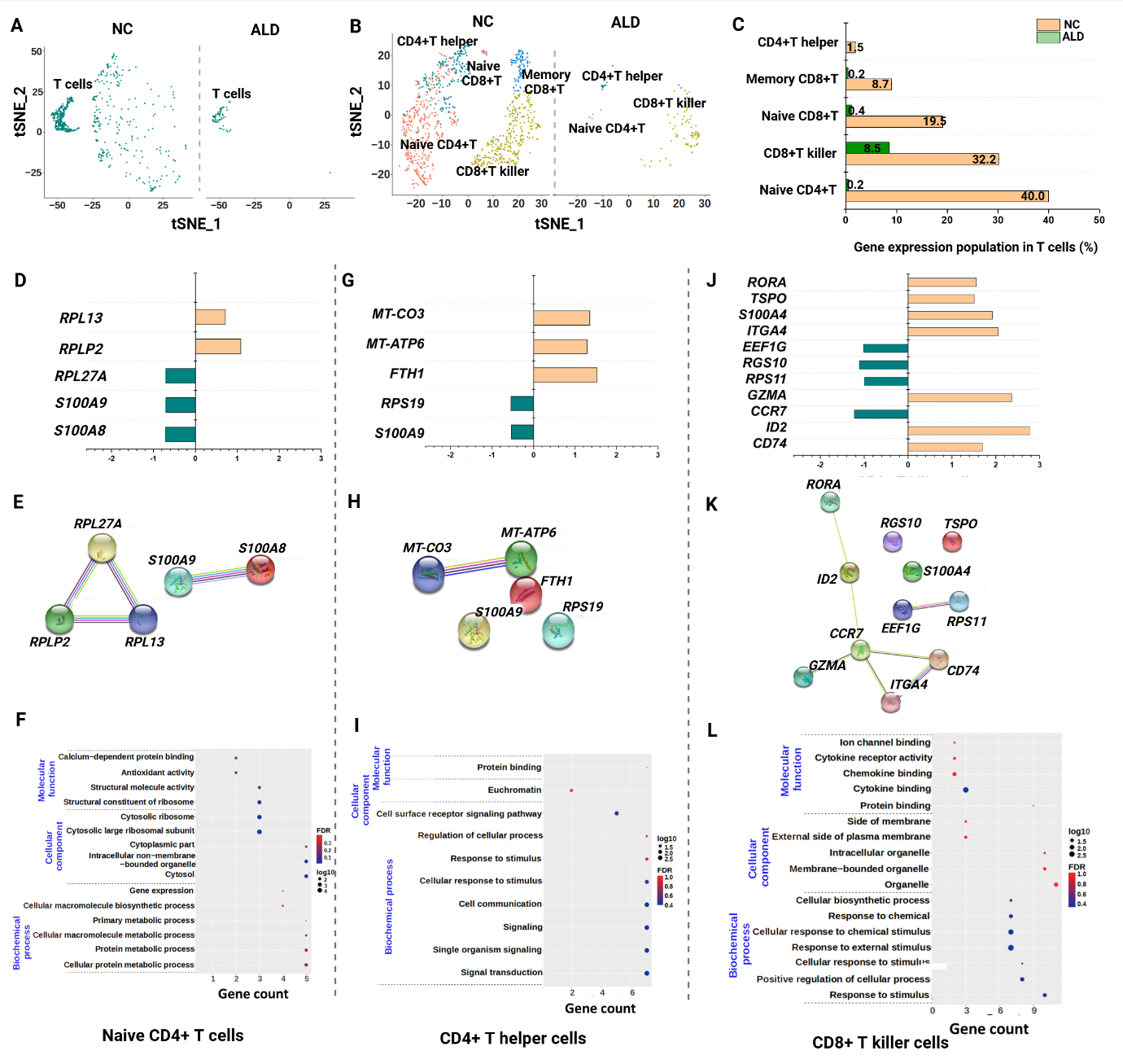


**Figure S4**. **Deep immunological profiling of T-cells** (**a**) tSNE visualization of total T-cells from NC and ALD-induced mouse groups. (**b**) Identification of T-cell subsets by applying the PhenoGraph clustering algorithm. (**c**) Population differences of each T-cell subset from NC and ALD-induced mouse groups. (**d, e, f**). The DEG expression, network profiling analysis and enrichment analysis GO of naïve CD4+T. (**g, h, i**). The DEG expression, network profiling analysis and enrichment analysis GO of CD4+T helper cells. (**j, k, l**). The DEG expression, network profiling analysis and enrichment analysis GO of CD8+T killer cells.

**Table S1**. Gene ontology enrichment analysis of Transitional B-cells

| **Category** | **Term** | **Count** | **%** | **Log10** | **PValue** | **Genes** | **Fold Enrichment** | **Bonferroni** | **FDR** |
| --- | --- | --- | --- | --- | --- | --- | --- | --- | --- |
| GOTERM_BP_ALL | GO:0050789~regulation of biological process | 11 | 91.6667 | -1.7739 | 0.016831 | PLAC8, ARPC2, CCL5, TXK, GZMA, TRBC1, TPR, NKG7, CD9, MALAT1, NRGN | 1.504313 | 1 | 1 |
| GOTERM_BP_ALL | GO:0050896~response to stimulus | 10 | 83.3333 | -1.6768 | 0.021046 | PLAC8, ARPC2, CCL5, TXK, GZMA, TRBC1, TPR, NKG7, CD9, NRGN | 1.673332 | 1 | 1 |
| GOTERM_BP_ALL | GO:0050794~regulation of cellular process | 10 | 83.3333 | -1.1917 | 0.064318 | PLAC8, ARPC2, CCL5, TXK, GZMA, TRBC1, TPR, CD9, MALAT1, NRGN | 1.452482 | 1 | 1 |
| GOTERM_BP_ALL | GO:0048522~positive regulation of cellular process | 9 | 75 | -2.6565 | 0.002205 | PLAC8, ARPC2, CCL5, TXK, GZMA, TRBC1, TPR, MALAT1, NRGN | 2.606227 | 0.91803 | 0.832879 |
| GOTERM_BP_ALL | GO:0009605~response to external stimulus | 7 | 58.3333 | -2.7619 | 0.00173 | PLAC8, CCL5, TXK, GZMA, TRBC1, NKG7, CD9 | 4.067939 | 0.859405 | 0.832879 |
| GOTERM_BP_ALL | GO:0006950~response to stress | 7 | 58.3333 | -1.9946 | 0.010125 | PLAC8, CCL5, TXK, TRBC1, TPR, NKG7, CD9 | 2.906478 | 0.99999 | 1 |
| GOTERM_CC_ALL | GO:0030425~dendrite | 3 | 25 |  | 0.05624 | ARPC2, MALAT1, NRGN | 6.94421 | 0.999962 | 1 |
|  |  |  |  |  |  |  |  |  |  |
| GOTERM_MF_ALL | GO:0005515~protein binding | 9 | 75 |  | 0.03494 | PLAC8, ARPC2, CCL5, GZMA, TRBC1, TPR, CD9, MALAT1, NRGN | 1.658905 | 0.980702 | 1 |
| GOTERM_MF_ALL | GO:0044877~macromolecular complex binding | 4 | 33.3333 |  | 0.03808 | ARPC2, TPR, CD9, MALAT1 | 4.548981 | 0.986559 | 1 |
| GOTERM_MF_ALL | GO:0042803~protein homodimerization activity | 3 | 25 |  | 0.056039 | CCL5, GZMA, TPR | 6.861615 | 0.998341 | 1 |
| GOTERM_MF_ALL | GO:0005102~receptor binding | 4 | 33.3333 |  | 0.062017 | ARPC2, CCL5, TRBC1, CD9 | 3.755635 | 0.99918 | 1 |

**Table S2**. Gene ontology enrichment analysis of Follicular B-cells

| **Category** | **Term** | **Count** | **%** | **PValue** | **Genes** | | **Fold Enrichment** | | | **Bonferroni** | **Benjamini** | **FDR** |
| --- | --- | --- | --- | --- | --- | --- | --- | --- | --- | --- | --- | --- |
| GOTERM_BP_ALL | GO:0042113~B cell activation | 11 | 55 | 1.40E-12 | CD79B, IGHM, CD74, MEF2C, SYK, IGKC, TRBC1, LEF1, IL7R, MS4A1, SIGLECG | | 24.52908 | | | 2.23E-09 | 2.23E-09 | 2.11E-09 |
| GOTERM_BP_ALL | GO:0046649~lymphocyte activation | 11 | 55 | 1.49E-09 | CD79B, IGHM, CD74, MEF2C, SYK, IGKC, TRBC1, LEF1, IL7R, MS4A1, SIGLECG | | 12.06791 | | | 2.38E-06 | 1.19E-06 | 1.12E-06 |
| GOTERM_BP_ALL | GO:0002250~adaptive immune response | 10 | 50 | 2.99E-09 | CD79B, IGHM, CD74, MEF2C, SYK, IGKC, TRBC1, LEF1, IL7R, SIGLECG | | 14.57908 | | | 4.76E-06 | 1.59E-06 | 1.50E-06 |
| GOTERM_BP_ALL | GO:0050853~B cell receptor signaling pathway | 7 | 35 | 2.43E-08 | CD79B, IGHM, MEF2C, SYK, IGKC, TRBC1, MS4A1 | | 33.76309 | | | 3.88E-05 | 6.25E-06 | 5.91E-06 |
| GOTERM_BP_ALL | GO:0002521~leukocyte differentiation | 9 | 45 | 2.86E-08 | CD79B, IGHM, CD74, MEF2C, SYK, IGKC, LEF1, IL7R, MS4A1 | | 14.90772 | | | 4.56E-05 | 6.25E-06 | 5.91E-06 |
| GOTERM_BP_ALL | GO:0051249~regulation of lymphocyte activation | 9 | 45 | 3.14E-08 | IGHM, CD74, MEF2C, SYK, IGKC, TRBC1, LEF1, IL7R, SIGLECG | | 14.72945 | | | 5.00E-05 | 6.25E-06 | 5.91E-06 |
| GOTERM_BP_ALL | GO:0030098~lymphocyte differentiation | 8 | 40 | 5.56E-08 | CD79B, IGHM, CD74, SYK, IGKC, LEF1, IL7R, MS4A1 | | 19.1247 | | | 8.87E-05 | 8.87E-06 | 8.39E-06 |
| GOTERM_CC_ALL | GO:0019815~B cell receptor complex | 3 | 15 | 1.31E-05 | CD79B, IGHM, SYK | | 495.9048 | | | 0.001824 | 6.23E-04 | 5.61E-04 |
| GOTERM_CC_ALL | GO:0019814~immunoglobulin complex | 5 | 25 | 4.00E-05 | CD79B, IGHM, SYK, IGKC, TRBC1 | | 23.51852 | | | 0.005551 | 0.001392 | 0.001252 |
| GOTERM_CC_ALL | GO:0022626~cytosolic ribosome | 4 | 20 | 1.14E-04 | RPL10, RPLP0, RPS21, RPS24 | | 39.22411 | | | 0.015688 | 0.003162 | 0.002844 |
| GOTERM_CC_ALL | GO:0009986~cell surface | 7 | 35 | 4.25E-04 | CD79B, IGHM, CD74, IGKC, TRBC1, IL7R, MS4A1 | | 6.154846 | | | 0.057374 | 0.009845 | 0.008854 |
| GOTERM_CC_ALL | GO:0044391~ribosomal subunit | 4 | 20 | 6.10E-04 | RPL10, RPLP0, RPS21, RPS24 | | 22.14567 | | | 0.08138 | 0.011668 | 0.010493 |
| GOTERM_CC_ALL | GO:0032991~macromolecular complex | 13 | 65 | 6.72E-04 | 2.400645 | 0.011668 | | 0.010493 |  |  |  |  |
| GOTERM_MF_DIRECT | GO:0003735~structural constituent of ribosome | 4 | 20 | 5.08E-04 | RPL10, RPLP0, RPS21, RPS24 | | 23.41749 | | | 0.039314 | 0.040098 | 0.040098 |
| GOTERM_MF_DIRECT | GO:0034987~immunoglobulin receptor binding | 3 | 15 | 0.010984 | IGHM, IGKC, TRBC1 | | 17.56312 | | | 0.582124 | 0.272932 | 0.272932 |
| GOTERM_MF_DIRECT | GO:0003823~antigen binding | 3 | 15 | 0.01299 | IGHM, IGKC, TRBC1 | | 16.08503 | | | 0.644038 | 0.272932 | 0.272932 |
| GOTERM_MF_DIRECT | GO:0042169~SH2 domain binding | 2 | 10 | 0.039249 | SYK, SIGLECG | | 47.08951 | | | 0.957709 | 0.555799 | 0.555799 |
| GOTERM_MF_DIRECT | GO:0004896~cytokine receptor activity | 2 | 10 | 0.049248 | CD74, IL7R | | 37.34686 | | | 0.981494 | 0.555799 | 0.555799 |
| GOTERM_MF_DIRECT | GO:0001228~transcriptional activator activity, RNA polymerase II transcription regulatory region sequence-specific binding | 3 | 15 | 0.075575 | MEF2C, LEF1, EBF1 | | 6.153743 | | | 0.997987 | 0.663381 | 0.663381 |

**Table S3**. Gene ontology enrichment analysis of Mature B-cells

| **Category** | **Term** | **Count** | **%** | **PValue** | **Genes** | **Fold Enrichment** | **Bonferroni** | **Benjamini** | **FDR** |
| --- | --- | --- | --- | --- | --- | --- | --- | --- | --- |
| GOTERM_BP_ALL | GO:0050789~regulation of biological process | 14 | 93.33333 | 0.00494 | CD83, LMO4, PLAUR, ATP1B1, H2-AA, COQ7, AKAP12, VPREB3, TIFA, IGLC1, S100A4, SOX4, ATF3, FGFR1 | 1.504313 | 0.998344 | 0.258298 | 0.2565 |
| GOTERM_BP_ALL | GO:0048522~positive regulation of cellular process | 13 | 86.66667 | 8.40E-06 | CD83, LMO4, PLAUR, ATP1B1, H2-AA, COQ7, AKAP12, TIFA, IGLC1, S100A4, SOX4, ATF3, FGFR1 | 2.957861 | 0.010806 | 0.010865 | 0.01079 |
| GOTERM_BP_ALL | GO:0050896~response to stimulus | 13 | 86.66667 | 0.00427 | CD83, PLAUR, ATP1B1, H2-AA, COQ7, AKAP12, VPREB3, TIFA, IGLC1, S100A4, SOX4, ATF3, FGFR1 | 1.709189 | 0.99607 | 0.251244 | 0.249495 |
| GOTERM_BP_ALL | GO:0050794~regulation of cellular process | 13 | 86.66667 | 0.01981 | CD83, LMO4, PLAUR, ATP1B1, H2-AA, COQ7, AKAP12, TIFA, IGLC1, S100A4, SOX4, ATF3, FGFR1 | 1.483606 | 1 | 0.323905 | 0.32165 |
| GOTERM_BP_ALL | GO:0051716~cellular response to stimulus | 10 | 66.66667 | 0.05527 | AKAP12, TIFA, PLAUR, IGLC1, S100A4, ATP1B1, COQ7, SOX4, ATF3, FGFR1 | 1.64568 | 1 | 0.516378 | 0.512784 |
| GOTERM_BP_ALL | GO:0048519~negative regulation of biological process | 9 | 60 | 0.01726 | VPREB3, CD83, LMO4, PLAUR, H2-AA, COQ7, SOX4, ATF3, FGFR1 | 2.160912 | 1 | 0.314357 | 0.312169 |
| GOTERM_CC_ALL | GO:0032991~macromolecular complex | 8 | 53.33333 | 0.09443 | LMO4, PLAUR, IGLC1, ATP1B1, H2-AA, SOX4, ATF3, FGFR1 | 1.772784 | 0.999997 | 1 | 1 |
| GOTERM_CC_ALL | GO:0044459~plasma membrane part | 7 | 46.66667 | 0.01412 | CD83, S100A6, PLAUR, IGLC1, ATP1B1, H2-AA, FGFR1 | 2.981513 | 0.838092 | 1 | 1 |
| GOTERM_CC_ALL | GO:0098552~side of membrane | 4 | 26.66667 | 0.02134 | CD83, S100A6, IGLC1, H2-AA | 6.076732 | 0.936743 | 1 | 1 |
| GOTERM_CC_ALL | GO:0009986~cell surface | 4 | 26.66667 | 0.05427 | CD83, PLAUR, IGLC1, H2-AA | 4.220466 | 0.999209 | 1 | 1 |
| GOTERM_CC_ALL | GO:0005667~transcription factor complex | 3 | 20 | 0.03971 | LMO4, SOX4, ATF3 | 8.732914 | 0.994408 | 1 | 1 |
| GOTERM_CC_ALL | GO:0009897~external side of plasma membrane | 3 | 20 | 0.07997 | CD83, IGLC1, H2-AA | 5.891938 | 0.999977 | 1 | 1 |
| GOTERM_MF_ALL | GO:0005488~binding | 13 | 86.66667 | 0.07669 | LMO4, PLAUR, ATP1B1, H2-AA, COQ7, AKAP12, TIFA, S100A6, IGLC1, S100A4, SOX4, ATF3, FGFR1 | 1.238531 | 0.999991 | 1 | 1 |
| GOTERM_MF_ALL | GO:0005515~protein binding | 12 | 80 | 0.00721 | AKAP12, LMO4, TIFA, S100A6, PLAUR, IGLC1, S100A4, ATP1B1, H2-AA, SOX4, ATF3, FGFR1 | 1.701441 | 0.650045 | 1 | 1 |
| GOTERM_MF_ALL | GO:0046983~protein dimerization activity | 4 | 26.66667 | 0.03752 | S100A6, ATP1B1, ATF3, FGFR1 | 4.792919 | 0.996091 | 1 | 1 |
| GOTERM_MF_ALL | GO:0042803~protein homodimerization activity | 3 | 20 | 0.09429 | S100A6, ATF3, FGFR1 | 5.278165 | 0.999999 | 1 | 1 |
| GOTERM_MF_ALL | GO:0048306~calcium-dependent protein binding | 2 | 13.33333 | 0.06082 | S100A6, S100A4 | 29.50641 | 0.999888 | 1 | 1 |

**Table S4**. Gene ontology enrichment analysis of Naïve B-cells

| **Category** | **Term** | **Count** | **%** |  | **PValue** | **Genes** | **Fold Enrichment** | **Bonferroni** | **Benjamini** | **FDR** |
| --- | --- | --- | --- | --- | --- | --- | --- | --- | --- | --- |
| GOTERM_BP_ALL | GO:0044237~cellular metabolic process | 6 | 100 |  | 0.0521 | CD74, GDA, MT-CO2, RPL37, RPL39, RPL17 | 1.805754 | 1 | 1 | 1 |
| GOTERM_BP_ALL | GO:0071704~organic substance metabolic process | 6 | 100 |  | 0.0718 | CD74, GDA, MT-CO2, RPL37, RPL39, RPL17 | 1.693127 | 1 | 1 | 1 |
| GOTERM_BP_ALL | GO:0044271~cellular nitrogen compound biosynthetic process | 5 | 83.333 |  | 0.0172 | CD74, MT-CO2, RPL37, RPL39, RPL17 | 3.250214 | 0.999998 | 1 | 1 |
| GOTERM_BP_ALL | GO:0044249~cellular biosynthetic process | 5 | 83.333 |  | 0.036 | CD74, MT-CO2, RPL37, RPL39, RPL17 | 2.661373 | 1 | 1 | 1 |
| GOTERM_BP_ALL | GO:0048522~positive regulation of cellular process | 5 | 83.333 |  | 0.0363 | CD74, GDA, MT-CO2, RPL37, RPL17 | 2.654491 | 1 | 1 | 1 |
| GOTERM_CC_ALL | GO:0005737~cytoplasm | 6 | 100 | -1.22 | 0.0601 | CD74, GDA, MT-CO2, RPL37, RPL39, RPL17 | 1.754823 | 0.996653 | 0.425128 | 0.406644 |
| GOTERM_CC_ALL | GO:0032991~macromolecular complex | 5 | 83.333 | -1.51 | 0.0311 | CD74, MT-CO2, RPL37, RPL39, RPL17 | 2.769976 | 0.945258 | 0.259977 | 0.248673 |
| GOTERM_CC_ALL | GO:0005829~cytosol | 4 | 66.667 | -1.23 | 0.0591 | GDA, RPL37, RPL39, RPL17 | 3.308395 | 0.996305 | 0.425128 | 0.406644 |
| GOTERM_CC_ALL | GO:0022625~cytosolic large ribosomal subunit | 3 | 50 | -3.98 | ###### | RPL37, RPL39, RPL17 | 153.1471 | 0.009556 | 0.009602 | 0.009184 |
| GOTERM_CC_ALL | GO:0015934~large ribosomal subunit | 3 | 50 | -3.42 | ###### | RPL37, RPL39, RPL17 | 80.10769 | 0.034528 | 0.011711 | 0.011201 |
| GOTERM_MF_ALL | GO:0003735~structural constituent of ribosome | 3 | 50 |  | ###### | RPL37, RPL39, RPL17 | 49.76216 | 0.066617 | 0.068905 | 0.068905 |
| GOTERM_MF_ALL | GO:0005198~structural molecule activity | 3 | 50 |  | 0.0136 | RPL37, RPL39, RPL17 | 13.05816 | 0.615316 | 0.474421 | 0.474421 |
| GOTERM_MF_ALL | GO:0019843~rRNA binding | 2 | 33.333 |  | 0.021 | RPL37, RPL17 | 78.68376 | 0.773733 | 0.490126 | 0.490126 |

**Table S5**. Gene ontology enrichment analysis of pro B-cells

| **Category** | **Term** | **Count** | **%** | **PValue** | **Genes** | **Fold Enrichment** | **Bonferroni** | **FDR** |
| --- | --- | --- | --- | --- | --- | --- | --- | --- |
| GOTERM_BP_DIRECT | GO:0070488~neutrophil aggregation | 2 | 33.33333 | 7.46E-04 | S100A9, S100A8 | 2232.667 | 0.035203 | 0.014176 |
| GOTERM_BP_DIRECT | GO:0035425~autocrine signaling | 2 | 33.33333 | 0.002238 | S100A9, S100A8 | 744.2222 | 0.10195 | 0.017006 |
| GOTERM_BP_DIRECT | GO:0002523~leukocyte migration involved in inflammatory response | 2 | 33.33333 | 0.004967 | S100A9, S100A8 | 334.9 | 0.212601 | 0.026965 |
| GOTERM_BP_DIRECT | GO:2001244~positive regulation of intrinsic apoptotic signaling pathway | 2 | 33.33333 | 0.009421 | S100A9, S100A8 | 176.2632 | 0.365134 | 0.044749 |
| GOTERM_BP_DIRECT | GO:0006919~activation of cysteine-type endopeptidase activity involved in apoptotic process | 2 | 33.33333 | 0.019751 | S100A9, S100A8 | 83.725 | 0.616153 | 0.075983 |
| GOTERM_BP_DIRECT | GO:0030593~neutrophil chemotaxis | 2 | 33.33333 | 0.019995 | S100A9, S100A8 | 82.69136 | 0.620729 | 0.075983 |
| GOTERM_BP_DIRECT | GO:0050729~positive regulation of inflammatory response | 2 | 33.33333 | 0.024395 | S100A9, S100A8 | 67.65657 | 0.694402 | 0.084274 |
| GOTERM_BP_DIRECT | GO:0006914~autophagy | 2 | 33.33333 | 0.047115 | S100A9, S100A8 | 34.70466 | 0.901386 | 0.127884 |
| GOTERM_BP_DIRECT | GO:0006954~inflammatory response | 2 | 33.33333 | 0.093358 | S100A9, S100A8 | 17.17436 | 0.990944 | 0.236508 |
| GOTERM_CC_DIRECT | GO:0045277~respiratory chain complex IV | 2 | 33.33333 | 0.002594 | MT-CO2, MT-CO3 | 641.7879 | 0.062882 | 0.047913 |
| GOTERM_CC_DIRECT | GO:0005751~mitochondrial respiratory chain complex IV | 2 | 33.33333 | 0.005654 | MT-CO2, MT-CO3 | 294.1528 | 0.132156 | 0.047913 |
| GOTERM_CC_DIRECT | GO:0005743~mitochondrial inner membrane | 3 | 50 | 0.00575 | MT-ATP6, MT-CO2, MT-CO3 | 20.32534 | 0.134245 | 0.047913 |
| GOTERM_CC_DIRECT | GO:0005739~mitochondrion | 3 | 50 | 0.065696 | MT-ATP6, MT-CO2, MT-CO3 | 5.632713 | 0.817103 | 0.32848 |
| GOTERM_MF_DIRECT | GO:0004129~cytochrome-c oxidase activity | 2 | 33.33333 | 0.005067 | MT-CO2, MT-CO3 | 328.2982 | 0.092005 | 0.065821 |
| GOTERM_MF_DIRECT | GO:0016209~antioxidant activity | 2 | 33.33333 | 0.006929 | S100A9, S100A8 | 239.9103 | 0.123746 | 0.065821 |
| GOTERM_MF_DIRECT | GO:0048306~calcium-dependent protein binding | 2 | 33.33333 | 0.025391 | S100A9, S100A8 | 64.97569 | 0.386557 | 0.160813 |
| **pre-pro B cells** | | | | | | | | |
| **Category** | **Term** | **Count** | **%** | **PValue** | **Genes** | **Fold Enrichment** | **Bonferroni** | **FDR** |
| GOTERM_BP_DIRECT | GO:0070488~neutrophil aggregation | 2 | 50 | 4.48E-04 | S100A9, S100A8 | 3349 | 0.019078 | 0.009627 |
| GOTERM_BP_DIRECT | GO:0035425~autocrine signaling | 2 | 50 | 0.001343 | S100A9, S100A8 | 1116.333 | 0.056156 | 0.011551 |
| GOTERM_BP_DIRECT | GO:0002523~leukocyte migration involved in inflammatory response | 2 | 50 | 0.002983 | S100A9, S100A8 | 502.35 | 0.120557 | 0.018325 |
| GOTERM_BP_DIRECT | GO:2001244~positive regulation of intrinsic apoptotic signaling pathway | 2 | 50 | 0.005663 | S100A9, S100A8 | 264.3947 | 0.216666 | 0.030438 |
| GOTERM_BP_DIRECT | GO:0030593~neutrophil chemotaxis | 2 | 50 | 0.012045 | S100A9, S100A8 | 124.037 | 0.406123 | 0.051794 |
| GOTERM_BP_DIRECT | GO:0050729~positive regulation of inflammatory response | 2 | 50 | 0.014709 | S100A9, S100A8 | 101.4848 | 0.471211 | 0.057497 |
| GOTERM_BP_DIRECT | GO:0006914~autophagy | 2 | 50 | 0.02854 | S100A9, S100A8 | 52.05699 | 0.712081 | 0.087659 |
| GOTERM_BP_DIRECT | GO:0006954~inflammatory response | 2 | 50 | 0.057106 | S100A9, S100A8 | 25.76154 | 0.920219 | 0.163705 |
| GOTERM_BP_DIRECT | GO:0002376~immune system process | 2 | 50 | 0.076497 | S100A9, S100A8 | 19.10076 | 0.967352 | 0.205585 |
| GOTERM_BP_DIRECT | GO:0006915~apoptotic process | 2 | 50 | 0.094223 | S100A9, S100A8 | 15.40951 | 0.985812 | 0.238328 |
| GOTERM_MF_DIRECT | GO:0016209~antioxidant activity | 2 | 50 | 0.004163 | S100A9, S100A8 | 359.8654 | 0.068457 | 0.055837 |
| GOTERM_MF_DIRECT | GO:0046872~metal ion binding | 4 | 100 | 0.00698 | GDA, MT-CO2, S100A9, S100A8 | 5.231479 | 0.112254 | 0.055837 |
| GOTERM_MF_DIRECT | GO:0048306~calcium-dependent protein binding | 2 | 50 | 0.015312 | S100A9, S100A8 | 97.46354 | 0.230738 | 0.081666 |
| **pre B cells** | | | | | | |  | |
| **Category** | **Term** | **Count** | **%** | **PValue** | **Genes** | **Fold Enrichment** | **Bonferroni** | **FDR** |
| GOTERM_BP_DIRECT | GO:0070488~neutrophil aggregation | 2 | 33.33333 | 7.46E-04 | S100A9, S100A8 | 2232.667 | 0.035203 | 0.01343 |
| GOTERM_BP_DIRECT | GO:0035425~autocrine signaling | 2 | 33.33333 | 0.002238 | S100A9, S100A8 | 744.2222 | 0.10195 | 0.016111 |
| GOTERM_BP_DIRECT | GO:0002523~leukocyte migration involved in inflammatory response | 2 | 33.33333 | 0.004967 | S100A9, S100A8 | 334.9 | 0.212601 | 0.025546 |
| GOTERM_BP_DIRECT | GO:0042776~mitochondrial ATP synthesis coupled proton transport | 2 | 33.33333 | 0.015826 | MT-ATP6, MT-ND1 | 104.6562 | 0.534993 | 0.059986 |
| GOTERM_BP_DIRECT | GO:0030593~neutrophil chemotaxis | 2 | 33.33333 | 0.019995 | S100A9, S100A8 | 82.69136 | 0.620729 | 0.059986 |
| GOTERM_BP_DIRECT | GO:0050729~positive regulation of inflammatory response | 2 | 33.33333 | 0.024395 | S100A9, S100A8 | 67.65657 | 0.694402 | 0.067556 |
| GOTERM_BP_DIRECT | GO:0032496~response to lipopolysaccharide | 2 | 33.33333 | 0.045678 | S100A9, S100A8 | 35.81818 | 0.893986 | 0.106009 |
| GOTERM_BP_DIRECT | GO:0006914~autophagy | 2 | 33.33333 | 0.047115 | S100A9, S100A8 | 34.70466 | 0.901386 | 0.106009 |
| GOTERM_BP_DIRECT | GO:0006954~inflammatory response | 2 | 33.33333 | 0.093358 | S100A9, S100A8 | 17.17436 | 0.990944 | 0.1977 |
| GOTERM_CC_DIRECT | GO:0005743~mitochondrial inner membrane | 4 | 66.66667 | 1.43E-04 | MT-ATP6, MT-CO2, MT-CO3, MT-ND1 | 27.10045 | 0.00356 | 0.003281 |
| GOTERM_CC_DIRECT | GO:0031966~mitochondrial membrane | 3 | 50 | 6.30E-04 | MT-CO2, MT-CO3, MT-ND1 | 62.29118 | 0.015642 | 0.00725 |
| GOTERM_CC_DIRECT | GO:0045277~respiratory chain complex IV | 2 | 33.33333 | 0.002594 | MT-CO2, MT-CO3 | 641.7879 | 0.062882 | 0.019891 |
| GOTERM_CC_DIRECT | GO:0005751~mitochondrial respiratory chain complex IV | 2 | 33.33333 | 0.005654 | MT-CO2, MT-CO3 | 294.1528 | 0.132156 | 0.028008 |
| GOTERM_CC_DIRECT | GO:0005739~mitochondrion | 4 | 66.66667 | 0.006089 | MT-ATP6, MT-CO2, MT-CO3, MT-ND1 | 7.510284 | 0.141597 | 0.028008 |
| GOTERM_MF_DIRECT | GO:0004129~cytochrome-c oxidase activity | 2 | 33.33333 | 0.005067 | MT-CO2, MT-CO3 | 328.2982 | 0.092005 | 0.065821 |
| GOTERM_MF_DIRECT | GO:0016209~antioxidant activity | 2 | 33.33333 | 0.006929 | S100A9, S100A8 | 239.9103 | 0.123746 | 0.065821 |
| GOTERM_MF_DIRECT | GO:0048306~calcium-dependent protein binding | 2 | 33.33333 | 0.025391 | S100A9, S100A8 | 64.97569 | 0.386557 | 0.160813 |

**Table S6**. Gene ontology enrichment analysis of Eosinophils

| **Category** | **Term** | **Count** | **%** | **PValue** | **Genes** | **Fold Enrichment** | **Bonferroni** | **FDR** |
| --- | --- | --- | --- | --- | --- | --- | --- | --- |
| GOTERM_BP_ALL | GO:0034641~cellular nitrogen compound metabolic process | 5 | 83.33333 | 0.051996 | RPLP1, MT-ND2, RPL39, S100A9, MT-ND1 | 2.405997 | 1 | 0.981133 |
| GOTERM_BP_ALL | GO:0006950~response to stress | 4 | 66.66667 | 0.073481 | IFITM1, RPL39, S100A9, MT-ND1 | 3.044882 | 1 | 0.981133 |
| GOTERM_BP_ALL | GO:0045087~innate immune response | 3 | 50 | 0.028519 | IFITM1, RPL39, S100A9 | 8.837668 | 0.99999 | 0.92088 |
| GOTERM_BP_ALL | GO:0043603~cellular amide metabolic process | 3 | 50 | 0.033257 | RPLP1, RPL39, S100A9 | 8.143802 | 0.999999 | 0.94544 |
| GOTERM_BP_ALL | GO:0043207~response to external biotic stimulus | 3 | 50 | 0.051239 | IFITM1, RPL39, S100A9 | 6.453176 | 1 | 0.981133 |
| GOTERM_BP_ALL | GO:0006952~defense response | 3 | 50 | 0.080125 | IFITM1, RPL39, S100A9 | 5.045571 | 1 | 0.981133 |
| GOTERM_CC_ALL | GO:0044446~intracellular organelle part | 6 | 100 | 0.017044 | IFITM1, RPLP1, MT-ND2, RPL39, S100A9, MT-ND1 | 2.257533 | 0.80801 | 0.186505 |
| GOTERM_CC_ALL | GO:0044444~cytoplasmic part | 6 | 100 | 0.018456 | IFITM1, RPLP1, MT-ND2, RPL39, S100A9, MT-ND1 | 2.22189 | 0.832757 | 0.186505 |
| GOTERM_CC_ALL | GO:0032991~macromolecular complex | 5 | 83.33333 | 0.031084 | IFITM1, RPLP1, MT-ND2, RPL39, MT-ND1 | 2.769976 | 0.951754 | 0.186505 |
| GOTERM_CC_ALL | GO:0030964~NADH dehydrogenase complex | 2 | 33.33333 | 0.012184 | MT-ND2, MT-ND1 | 136.1307 | 0.691766 | 0.186505 |
| GOTERM_CC_ALL | GO:0005747~mitochondrial respiratory chain complex I | 2 | 33.33333 | 0.012184 | MT-ND2, MT-ND1 | 136.1307 | 0.691766 | 0.186505 |
| GOTERM_CC_ALL | GO:0022625~cytosolic large ribosomal subunit | 2 | 33.33333 | 0.016219 | RPLP1, RPL39 | 102.098 | 0.791921 | 0.186505 |
| GOTERM_MF_ALL | GO:0008137~NADH dehydrogenase (ubiquinone) activity | 2 | 33.33333 | 0.005204 | MT-ND2, MT-ND1 | 306.8667 | 0.153775 | 0.064954 |
| GOTERM_MF_ALL | GO:0016655~oxidoreductase activity, acting on NAD(P)H, quinone or similar compound as acceptor | 2 | 33.33333 | 0.00931 | MT-ND2, MT-ND1 | 171.2744 | 0.25867 | 0.072151 |
| GOTERM_MF_ALL | GO:0003735~structural constituent of ribosome | 2 | 33.33333 | 0.039593 | RPLP1, RPL39 | 39.80973 | 0.725479 | 0.204562 |

**Table S7**. Gene ontology enrichment analysis of Neutrophils

| **Category** | **Term** | **Count** | **%** | **PValue** | **Genes** | **Fold Enrichment** | **Bonferroni** | **FDR** |
| --- | --- | --- | --- | --- | --- | --- | --- | --- |
| GOTERM_BP_ALL | GO:0044267~cellular protein metabolic process | 10 | 62.5 | 0.005982 | RPS18, IL1B, NGP, FN1, CSTDC4, STFA1, STFA2L1, CAMP, S100A10, CSTDC5 | 2.379274 | 0.999387 | 0.226376 |
| GOTERM_BP_ALL | GO:0048731~system development | 10 | 62.5 | 0.007527 | LGALS1, IL1B, NGP, FN1, CSTDC4, STFA1, STFA2L1, CAMP, S100A10, CSTDC5 | 2.304059 | 0.99991 | 0.269613 |
| GOTERM_BP_ALL | GO:0019538~protein metabolic process | 10 | 62.5 | 0.016344 | RPS18, IL1B, NGP, FN1, CSTDC4, STFA1, STFA2L1, CAMP, S100A10, CSTDC5 | 2.061506 | 1 | 0.436199 |
| GOTERM_BP_ALL | GO:0060255~regulation of macromolecule metabolic process | 10 | 62.5 | 0.035549 | IL1B, NGP, FN1, CSTDC4, CRIP1, STFA1, STFA2L1, CAMP, S100A10, CSTDC5 | 1.83378 | 1 | 0.551915 |
| GOTERM_BP_ALL | GO:0032502~developmental process | 10 | 62.5 | 0.05139 | LGALS1, IL1B, NGP, FN1, CSTDC4, STFA1, STFA2L1, CAMP, S100A10, CSTDC5 | 1.730229 | 1 | 0.699245 |
| GOTERM_BP_ALL | GO:0019222~regulation of metabolic process | 10 | 62.5 | 0.056323 | IL1B, NGP, FN1, CSTDC4, CRIP1, STFA1, STFA2L1, CAMP, S100A10, CSTDC5 | 1.704844 | 1 | 0.726232 |
| GOTERM_CC_ALL | GO:0005576~extracellular region | 14 | 87.5 | 1.01E-09 | LYZ2, FN1, CXCL2, STFA1, STFA2L1, LGALS1, NGP, IL1B, S100A4, WFDC17, CSTDC4, CAMP, S100A10, CSTDC5 | 6.014686 | 1.14E-07 | 5.48E-08 |
| GOTERM_CC_ALL | GO:0005615~extracellular space | 13 | 81.25 | 4.38E-10 | LYZ2, FN1, CXCL2, STFA1, STFA2L1, LGALS1, NGP, IL1B, WFDC17, CSTDC4, CAMP, S100A10, CSTDC5 | 7.933779 | 4.95E-08 | 4.77E-08 |
| GOTERM_CC_ALL | GO:0044444~cytoplasmic part | 13 | 81.25 | 0.00632 | LYZ2, FN1, STFA1, STFA2L1, LGALS1, RPS18, NGP, IL1B, S100A4, CSTDC4, CAMP, S100A10, CSTDC5 | 1.805286 | 0.511529 | 0.114821 |
| GOTERM_CC_ALL | GO:0043227~membrane-bounded organelle | 13 | 81.25 | 0.074812 | LYZ2, FN1, STFA1, STFA2L1, LGALS1, RPS18, NGP, IL1B, S100A4, CSTDC4, CAMP, S100A10, CSTDC5 | 1.384727 | 0.999847 | 0.741315 |
| GOTERM_CC_ALL | GO:0005829~cytosol | 7 | 43.75 | 0.062947 | LGALS1, RPS18, IL1B, CSTDC4, STFA1, STFA2L1, CSTDC5 | 2.171134 | 0.999355 | 0.741315 |
| GOTERM_CC_ALL | GO:1904090~peptidase inhibitor complex | 4 | 25 | 8.61E-08 | CSTDC4, STFA1, STFA2L1, CSTDC5 | 400.5385 | 9.72E-06 | 2.34E-06 |
| GOTERM_MF_ALL | GO:0061134~peptidase regulator activity | 7 | 43.75 | 4.80E-08 | NGP, FN1, WFDC17, CSTDC4, STFA1, STFA2L1, CSTDC5 | 29.18569 | 3.99E-06 | 3.70E-06 |
| GOTERM_MF_ALL | GO:0030234~enzyme regulator activity | 7 | 43.75 | 1.34E-04 | NGP, FN1, WFDC17, CSTDC4, STFA1, STFA2L1, CSTDC5 | 7.390138 | 0.011037 | 0.001287 |
| GOTERM_MF_ALL | GO:0098772~molecular function regulator | 7 | 43.75 | 7.37E-04 | NGP, FN1, WFDC17, CSTDC4, STFA1, STFA2L1, CSTDC5 | 5.402582 | 0.059342 | 0.006304 |
| GOTERM_MF_ALL | GO:0004866~endopeptidase inhibitor activity | 6 | 37.5 | 7.24E-07 | NGP, WFDC17, CSTDC4, STFA1, STFA2L1, CSTDC5 | 30.55088 | 6.01E-05 | 1.53E-05 |
| GOTERM_MF_ALL | GO:0061135~endopeptidase regulator activity | 6 | 37.5 | 9.95E-07 | NGP, WFDC17, CSTDC4, STFA1, STFA2L1, CSTDC5 | 28.64938 | 8.25E-05 | 1.53E-05 |
| GOTERM_MF_ALL | GO:0002020~protease binding | 5 | 31.25 | 1.32E-05 | FN1, CSTDC4, STFA1, STFA2L1, CSTDC5 | 30.60505 | 0.001092 | 1.69E-04 |

**Table S8**. Gene ontology enrichment analysis of Classical Monocytes

| **Category** | **Term** | **Count** | **%** | **PValue** | **Genes** | **Fold Enrichment** | **Bonferroni** | **FDR** |
| --- | --- | --- | --- | --- | --- | --- | --- | --- |
| GOTERM_BP_DIRECT | GO:0042776~mitochondrial ATP synthesis coupled proton transport | 4 | 26.66667 | 1.09E-05 | MT-ATP6, MT-ND4, MT-ND2, MT-ND1 | 83.725 | 0.001017 | 7.32E-04 |
| GOTERM_BP_DIRECT | GO:0015990~electron transport coupled proton transport | 2 | 13.33333 | 0.003479 | MT-ND4, MT-CYTB | 535.84 | 0.276838 | 0.062624 |
| GOTERM_BP_DIRECT | GO:0042773~ATP synthesis coupled electron transport | 2 | 13.33333 | 0.004868 | MT-ND4, MT-CO2 | 382.7429 | 0.364786 | 0.073014 |
| GOTERM_BP_DIRECT | GO:0006412~cytoplasmictranslation | 3 | 20 | 0.019451 | RPS8, RPL37, FAU | 12.83962 | 0.839074 | 0.194515 |
| GOTERM_BP_DIRECT | GO:0030334~regulation of cell migration | 2 | 13.33333 | 0.081752 | TMSB4X, TMSB10 | 21.96066 | 0.999641 | 0.668876 |
|  |  |  |  |  |  |  |  |  |
| GOTERM_CC_DIRECT | GO:0005743~mitochondrial inner membrane | 7 | 46.66667 | 5.47E-07 | MT-ATP6, MT-ND4, MT-CO2, MT-CYTB, MT-CO3, MT-ND2, MT-ND1 | 18.97031 | 4.10E-05 | 1.89E-05 |
| GOTERM_CC_DIRECT | GO:0005747~mitochondrial respiratory chain complex I | 3 | 20 | 5.08E-04 | MT-ND4, MT-ND2, MT-ND1 | 83.0549 | 0.037383 | 0.008761 |
| GOTERM_CC_DIRECT | GO:0022626~cytosolic ribosome | 3 | 20 | 0.001276 | RPS8, RPL37, FAU | 52.29383 | 0.091323 | 0.014675 |
| GOTERM_CC_DIRECT | GO:0022627~cytosolic small ribosomal subunit | 2 | 13.33333 | 0.031918 | RPS8, FAU | 57.62993 | 0.912214 | 0.220231 |
|  |  |  |  |  |  |  |  |  |
| GOTERM_MF_DIRECT | GO:0008137~NADH dehydrogenase (ubiquinone) activity | 3 | 20 | 1.42E-04 | MT-ND4, MT-ND2, MT-ND1 | 155.9417 | 0.005103 | 0.005116 |
| GOTERM_MF_DIRECT | GO:0003735~structural constituent of ribosome | 3 | 20 | 0.008267 | RPS8, RPL37, FAU | 20.12151 | 0.258321 | 0.125122 |
| GOTERM_MF_DIRECT | GO:0003785~actin monomer binding | 2 | 13.33333 | 0.020018 | TMSB4X, TMSB10 | 92.40988 | 0.517113 | 0.144132 |

**Table S9**. Gene ontology enrichment analysis of Macrophages

| **Category** | **Term** | **Count** | **%** | **PValue** | **Genes** | **Fold Enrichment** | **Bonferroni** | **FDR** |
| --- | --- | --- | --- | --- | --- | --- | --- | --- |
| GOTERM_BP_DIRECT | GO:0002181~cytoplasmic translation | 4 | 40 | 6.46E-06 | RPLP1, RPL37A, RPL13, FAU | 92.38621 | 3.75E-04 | 3.75E-04 |
| GOTERM_BP_DIRECT | GO:0006412~translation | 3 | 30 | 0.0081 | RPL37A, RPL13, FAU | 19.25942 | 0.376062 | 0.234896 |
| GOTERM_CC_DIRECT | GO:0022626~cytosolic ribosome | 4 | 40 | 4.45E-06 | RPLP1, RPL37A, RPL13, FAU | 104.5877 | 1.07E-04 | 9.35E-05 |
| GOTERM_CC_DIRECT | GO:0005840~ribosome | 4 | 40 | 6.10E-05 | RPLP1, RPL37A, RPL13, FAU | 43.66804 | 0.001464 | 6.41E-04 |
| GOTERM_CC_DIRECT | GO:0022625~cytosolic large ribosomal subunit | 3 | 30 | 3.60E-04 | RPLP1, RPL37A, RPL13 | 93.43676 | 0.008614 | 0.002523 |
| GOTERM_CC_DIRECT | GO:0045277~respiratory chain complex IV | 2 | 20 | 0.004666 | MT-CO2, MT-CO3 | 385.0727 | 0.106168 | 0.024495 |
| GOTERM_CC_DIRECT | GO:0005751~mitochondrial respiratory chain complex IV | 2 | 20 | 0.010155 | MT-CO2, MT-CO3 | 176.4917 | 0.217261 | 0.042649 |
| GOTERM_CC_DIRECT | GO:0031966~mitochondrial membrane | 2 | 20 | 0.069978 | MT-CO2, MT-CO3 | 24.91647 | 0.824676 | 0.244922 |
| GOTERM_CC_DIRECT | GO:0005829~cytosol | 5 | 50 | 0.082952 | GDA, RPLP1, RPL37A, RPL13, S100A8 | 2.525519 | 0.874856 | 0.248856 |
| GOTERM_MF_DIRECT | GO:0003735~structural constituent of ribosome | 4 | 40 | 7.77E-05 | RPLP1, RPL37A, RPL13, FAU | 40.24301 | 0.002327 | 0.00233 |
| GOTERM_MF_DIRECT | GO:0004129~cytochrome-c oxidase activity | 2 | 20 | 0.009103 | MT-CO2, MT-CO3 | 196.9789 | 0.239926 | 0.136544 |
| GOTERM_MF_DIRECT | GO:0046872~metal ion binding | 5 | 50 | 0.074355 | GDA, FTH1, RPL37A, MT-CO2, S100A8 | 2.615739 | 0.901524 | 0.743552 |

**Table S10**. Gene ontology enrichment analysis of Naïve CD4+T-cells

| **Category** | **Term** | **Count** | **%** |  | **PValue** | **Genes** | **Fold Enrichment** | **Bonferroni** | **FDR** |
| --- | --- | --- | --- | --- | --- | --- | --- | --- | --- |
| GOTERM_BP_ALL | GO:0044267~cellular protein metabolic process | 5 | 100 | 2.322629 | 0.004757 | RPL27A, RPLP2, RPL13, S100A9, S100A8 | 3.806837937 | 0.730560561 | 0.178743 |
| GOTERM_BP_ALL | GO:0019538~protein metabolic process | 5 | 100 | 2.073523 | 0.008443 | RPL27A, RPLP2, RPL13, S100A9, S100A8 | 3.298410042 | 0.902857651 | 0.202924 |
| GOTERM_BP_ALL | GO:0044260~cellular macromolecule metabolic process | 5 | 100 | 1.38078 | 0.041612 | RPL27A, RPLP2, RPL13, S100A9, S100A8 | 2.21388452 | 0.999991608 | 0.332754 |
| GOTERM_BP_ALL | GO:0044238~primary metabolic process | 5 | 100 | 1.006168 | 0.09859 | RPL27A, RPLP2, RPL13, S100A9, S100A8 | 1.78449837 | 1 | 0.398909 |
| GOTERM_BP_ALL | GO:0034645~cellular macromolecule biosynthetic process | 4 | 80 | 1.23391 | 0.058357 | RPL27A, RPLP2, RPL13, S100A9 | 3.042531841 | 0.999999934 | 0.392917 |
| GOTERM_BP_ALL | GO:0010467~gene expression | 4 | 80 | 1.070321 | 0.085051 | RPL27A, RPLP2, RPL13, S100A9 | 2.65070612 | 1 | 0.396816 |
| GOTERM_CC_ALL | GO:0005829~cytosol | 5 | 100 | 2.78333 | 0.001647 | RPL27A, RPLP2, RPL13, S100A9, S100A8 | 4.962592328 | 0.071488035 | 0.008705 |
| GOTERM_CC_ALL | GO:0043232~intracellular non-membrane-bounded organelle | 5 | 100 | 2.561667 | 0.002744 | RPL27A, RPLP2, RPL13, S100A9, S100A8 | 4.368288591 | 0.116297849 | 0.011451 |
| GOTERM_CC_ALL | GO:0044444~cytoplasmic part | 5 | 100 | 1.387043 | 0.041016 | RPL27A, RPLP2, RPL13, S100A9, S100A8 | 2.221890335 | 0.848118502 | 0.126467 |
| GOTERM_CC_ALL | GO:0022625~cytosolic large ribosomal subunit | 3 | 60 | 4.202376 | 6.28E-05 | RPL27A, RPLP2, RPL13 | 183.7764706 | 0.002819921 | 0.002322 |
| GOTERM_CC_ALL | GO:0022626~cytosolic ribosome | 3 | 60 | 3.722286 | 1.90E-04 | RPL27A, RPLP2, RPL13 | 105.9050847 | 0.00849409 | 0.002837 |
| GOTERM_MF_ALL | GO:0003735~structural constituent of ribosome | 3 | 60 | 3.22581 | 5.95E-04 | RPL27A, RPLP2, RPL13 | 59.71459459 | 0.02060035 | 0.020809 |
| GOTERM_MF_ALL | GO:0005198~structural molecule activity | 3 | 60 | 2.078627 | 0.008344 | RPL27A, RPLP2, RPL13 | 15.66978723 | 0.254175093 | 0.14602 |
| GOTERM_MF_ALL | GO:0016209~antioxidant activity | 2 | 40 | 1.693205 | 0.020267 | S100A9, S100A8 | 78.34893617 | 0.511609776 | 0.181082 |
| GOTERM_MF_ALL | GO:0048306~calcium-dependent protein binding | 2 | 40 | 1.684133 | 0.020695 | S100A9, S100A8 | 76.71666667 | 0.519019308 | 0.181082 |

**Table S11**. Gene ontology enrichment analysis of CD4+T helper cells

| **Category** | **Term** | **Count** | **%** | **PValue** | **Genes** | **Fold Enrichment** | **Bonferroni** | **FDR** |
| --- | --- | --- | --- | --- | --- | --- | --- | --- |
| GOTERM_BP_ALL | GO:0006955~immune response | 3 | 60 | 0.05804 | RPS19, FTH1, S100A9 | 5.671367 | 1 | 1 |
| GOTERM_BP_ALL | GO:0061844~antimicrobial humoral immune response mediated by antimicrobial peptide | 2 | 40 | 0.029903 | RPS19, S100A9 | 52.90738 | 0.999999 | 1 |
| GOTERM_BP_ALL | GO:0050729~positive regulation of inflammatory response | 2 | 40 | 0.032677 | RPS19, S100A9 | 48.36319 | 1 | 1 |
| GOTERM_BP_ALL | GO:0019730~antimicrobial humoral response | 2 | 40 | 0.037026 | RPS19, S100A9 | 42.61189 | 1 | 1 |
| GOTERM_BP_ALL | GO:0097529~myeloid leukocyte migration | 2 | 40 | 0.046266 | RPS19, S100A9 | 33.97931 | 1 | 1 |
| GOTERM_BP_ALL | GO:0030595~leukocyte chemotaxis | 2 | 40 | 0.047049 | RPS19, S100A9 | 33.40339 | 1 | 1 |
| GOTERM_CC_ALL | GO:0044446~intracellular organelle part | 5 | 100 | 0.038486 | MT-ATP6, RPS19, FTH1, MT-CO3, S100A9 | 2.257533 | 0.970759 | 0.639625 |
| GOTERM_CC_ALL | GO:0044444~cytoplasmic part | 5 | 100 | 0.041016 | MT-ATP6, RPS19, FTH1, MT-CO3, S100A9 | 2.22189 | 0.976932 | 0.639625 |
| GOTERM_CC_ALL | GO:0005739~mitochondrion | 3 | 60 | 0.047176 | MT-ATP6, FTH1, MT-CO3 | 6.340335 | 0.987084 | 0.639625 |
| GOTERM_CC_ALL | GO:0098800~inner mitochondrial membrane protein complex | 2 | 40 | 0.027183 | MT-ATP6, MT-CO3 | 58.26014 | 0.916288 | 0.639625 |
| GOTERM_CC_ALL | GO:0098798~mitochondrial protein complex | 2 | 40 | 0.033939 | MT-ATP6, MT-CO3 | 46.54302 | 0.955288 | 0.639625 |
| GOTERM_CC_ALL | GO:0044455~mitochondrial membrane part | 2 | 40 | 0.049749 | MT-ATP6, MT-CO3 | 31.55758 | 0.989873 | 0.639625 |
| GOTERM_MF_ALL | GO:0015078~hydrogen ion transmembrane transporter activity | 2 | 40 | 0.021337 | MT-ATP6, MT-CO3 | 74.39192 | 0.743019 | 0.846817 |
| GOTERM_MF_ALL | GO:0015399~primary active transmembrane transporter activity | 2 | 40 | 0.026883 | MT-ATP6, MT-CO3 | 58.9184 | 0.820363 | 0.846817 |
| GOTERM_MF_ALL | GO:0015077~monovalent inorganic cation transmembrane transporter activity | 2 | 40 | 0.071028 | MT-ATP6, MT-CO3 | 21.91905 | 0.990358 | 1 |
| GOTERM_MF_ALL | GO:0022804~active transmembrane transporter activity | 2 | 40 | 0.078407 | MT-ATP6, MT-CO3 | 19.79785 | 0.994166 | 1 |

**Table S12**. Gene ontology enrichment analysis of CD8+T killer cells

| **Category** | **Term** | **Count** | **%** | **PValue** | **Genes** | **Fold Enrichment** | **Bonferroni** | **FDR** |
| --- | --- | --- | --- | --- | --- | --- | --- | --- |
| GOTERM_BP_ALL | GO:0050896~response to stimulus | 10 | 90.90909 | 0.021046 | EEF1G, CD74, ITGA4, GZMA, ID2, RGS10, TSPO, S100A4, CCR7, RORA | 1.673332 | 1 | 0.370055 |
| GOTERM_BP_ALL | GO:0048522~positive regulation of cellular process | 8 | 72.72727 | 0.013835 | CD74, ITGA4, GZMA, ID2, TSPO, S100A4, CCR7, RORA | 2.316646 | 1 | 0.353325 |
| GOTERM_BP_ALL | GO:0051716~cellular response to stimulus | 8 | 72.72727 | 0.084562 | CD74, ITGA4, ID2, RGS10, TSPO, S100A4, CCR7, RORA | 1.675601 | 1 | 0.566752 |
| GOTERM_BP_ALL | GO:0009605~response to external stimulus | 7 | 63.63636 | 0.00173 | EEF1G, CD74, GZMA, ID2, TSPO, CCR7, RORA | 4.067939 | 0.907853 | 0.332257 |
| GOTERM_BP_ALL | GO:0070887~cellular response to chemical stimulus | 7 | 63.63636 | 0.003476 | CD74, ITGA4, ID2, RGS10, TSPO, CCR7, RORA | 3.570013 | 0.991728 | 0.332257 |
| GOTERM_BP_ALL | GO:0042221~response to chemical | 7 | 63.63636 | 0.02032 | CD74, ITGA4, ID2, RGS10, TSPO, CCR7, RORA | 2.529539 | 1 | 0.370055 |
| GOTERM_BP_ALL | GO:0044249~cellular biosynthetic process | 7 | 63.63636 | 0.057657 | EEF1G, CD74, ID2, TSPO, CCR7, RORA, RPS11 | 2.032321 | 1 | 0.489001 |
| GOTERM_CC_ALL | GO:0043226~organelle | 11 | 100 | 0.014147 | EEF1G, CD74, ITGA4, GZMA, ID2, RGS10, TSPO, S100A4, CCR7, RORA, RPS11 | 1.530683 | 0.800107 | 1 |
| GOTERM_CC_ALL | GO:0043227~membrane-bounded organelle | 10 | 90.90909 | 0.03887 | EEF1G, CD74, ITGA4, GZMA, ID2, RGS10, TSPO, S100A4, CCR7, RORA | 1.549345 | 0.988666 | 1 |
| GOTERM_CC_ALL | GO:0043229~intracellular organelle | 10 | 90.90909 | 0.073152 | EEF1G, CD74, GZMA, ID2, RGS10, TSPO, S100A4, CCR7, RORA, RPS11 | 1.428375 | 0.999813 | 1 |
| GOTERM_CC_ALL | GO:0009897~external side of plasma membrane | 3 | 27.27273 | 0.043218 | CD74, ITGA4, CCR7 | 8.034461 | 0.99321 | 1 |
| GOTERM_CC_ALL | GO:0098552~side of membrane | 3 | 27.27273 | 0.068516 | CD74, ITGA4, CCR7 | 6.21484 | 0.999671 | 1 |
| GOTERM_MF_ALL | GO:0005515~protein binding | 9 | 81.81818 | 0.091459 | CD74, ITGA4, GZMA, ID2, RGS10, TSPO, S100A4, CCR7, RORA | 1.508095 | 0.999988 | 1 |
| GOTERM_MF_ALL | GO:0019955~cytokine binding | 3 | 27.27273 | 0.002659 | CD74, ITGA4, CCR7 | 34.63072 | 0.269637 | 0.313796 |
| GOTERM_MF_ALL | GO:0019956~chemokine binding | 2 | 18.18182 | 0.018318 | ITGA4, CCR7 | 98.45989 | 0.887135 | 1 |
| GOTERM_MF_ALL | GO:0004896~cytokine receptor activity | 2 | 18.18182 | 0.053017 | CD74, CCR7 | 33.47636 | 0.998384 | 1 |
| GOTERM_MF_ALL | GO:0044325~ion channel binding | 2 | 18.18182 | 0.079069 | ID2, TSPO | 22.16978 | 0.99994 | 1 |
